# A Genotypic Comparison Reveals That the Improvement in Nitrogen Remobilization Efficiency in Oilseed Rape Leaves Is Related to Specific Patterns of Senescence-Associated Protease Activities and Phytohormones

**DOI:** 10.3389/fpls.2019.00046

**Published:** 2019-02-04

**Authors:** Marine Poret, Balakumaran Chandrasekar, Renier A. L. van der Hoorn, Sylvain Déchaumet, Alain Bouchereau, Tae-Hwan Kim, Bok-Rye Lee, Flavien Macquart, Ikuko Hara-Nishimura, Jean-Christophe Avice

**Affiliations:** ^1^Université de Caen Normandie, UMR INRA–UCBN 950 Ecophysiologie Végétale, Agronomie & Nutritions N.C.S., FED 4277 Normandie Végétal, Caen, France; ^2^Plant Chemetics Laboratory, Department of Plant Sciences, University of Oxford, Oxford, United Kingdom; ^3^Plant Chemetics Laboratory, Max Planck Institute for Plant Breeding Research, Cologne, Germany; ^4^INRA, UMR 1349 Institut de Génétique, Environnement et Protection des Plantes, INRA, Agrocampus Ouest, Université de Rennes 1, Rennes, France; ^5^Department of Animal Science, Institute of Agricultural Science and Technology, College of Agriculture and Life Sciences, Chonnam National University, Gwangju, South Korea; ^6^Laboratory of Plant Cell Biology, Faculty of Science and Engineering, Konan University Okamoto, Kobe, Japan

**Keywords:** *Brassica napus* L., nitrogen remobilization efficiency, senescence, protease activity, regulation, phytohormones

## Abstract

Oilseed rape (*Brassica napus* L.) is an oleoproteaginous crop characterized by low N use efficiency (NUE) that is mainly related to a weak Nitrogen Remobilization Efficiency (NRE) during the sequential leaf senescence of the vegetative stages. Based on the hypothesis that proteolysis efficiency is crucial for the improvement of leafNRE, our objective was to characterize key senescence-associated proteolytic mechanisms of two genotypes (Ténor and Samouraï) previously identified with contrasting NREs. To reach this goal, biochemical changes, protease activities and phytohormone patterns were studied in mature leaves undergoing senescence in two genotypes with contrasting NRE cultivated in a greenhouse under limiting or ample nitrate supply. The genotype with the higher NRE (Ténor) possessed enhanced senescence processes in response to nitrate limitation, and this led to greater degradation of soluble proteins compared to the other genotype (Samouraï). This efficient proteolysis is associated with (i) an increase in serine and cysteine protease (CP) activities and (ii) the appearance of new CP activities (RD21-like, SAG12-like, RD19-like, cathepsin-B, XBCP3-like and aleurain-like proteases) during senescence induced by N limitation. Compared to Samouraï, Ténor has a higher hormonal ratio ([salicylic acid] + [abscisic acid])/([cytokinins]) that promotes senescence, particularly under low N conditions, and this is correlated with the stronger protein degradation and serine/CP activities observed during senescence. Short statement: The improvement in N recycling during leaf senescence in a genotype of *Brassica napus* L. characterized by a high nitrogen remobilization efficiency is related to a high phytohormonal ratio ([salicylic acid] + [abscisic acid])/([cytokinins]) that promotes leaf senescence and is correlated with an increase or the induction of specific serine and cysteine protease activities.

## Introduction

The increase in crop productivity during the last five decades is due particularly to an increase in nitrogen (N) fertilizer inputs (Glass, 2003) in relation to improvements in genetic performance and culture practices. Nevertheless, N fertilizers represent the most costly inputs in crop production (Rothstein, 2007) and their widespread use substantially increases the risk of N pollution. That is why a reduction in N inputs has become a priority for reducing the economic environmental costs in a context of sustainable agriculture (Behrens et al., 2001).

While oilseed rape (*Brassica napus* L.) is the dominant oleoproteaginous crop in northern Europe, it has a high demand for N fertilizers (160–250 kg N ha^-1^ year^-1^) to attain a satisfactory seed yield (Rathke et al., 2005). Despite a considerable capacity to absorb N (Laîné et al., 1993), the N use efficiency (NUE) of winter oilseed rape is lower than other crop plants such as wheat or barley (Sylvester-Bradley and Kindred, 2009). Indeed, only 50% of the N originating from fertilizers is recovered in the seeds while a significant proportion is returned to the environment (Schjoerring et al., 1995) leading to a negative economic and agro-environmental balance for oilseed rape. Several studies have proved that this weak NUE is mainly due to a poor N Remobilization Efficiency (NRE) during the ‘sequential’ leaf senescence that occurs in the vegetative stages and during the transition between vegetative and reproductive phases of development in oilseed rape (Malagoli et al., 2005a,b; Gombert et al., 2006; Avice and Etienne, 2014). Indeed, a recent screening of NUE, NRE and senescence processes in ten genotypes of oilseed rape defined 4 genotypic profiles with different behaviors during vegetative-stage senescence that were associated with N limitation (Girondé et al., 2015). These authors showed that genotypes with the highest N use efficiency were also characterized by an efficient NRE.

Sequential senescence gradually affects older leaves along the axis of the plant and leads to nutrient remobilization from the source leaves to the young leaves and other sink organs (Guiboileau et al., 2010; Avice and Etienne, 2014). Leaf senescence is tightly linked to global plant productivity and the seed crop yield (Wu et al., 2012; Gregersen et al., 2013), especially in response to low N fertilization. This is a complex process controlled by endogenous and environmental factors (Guo and Gan, 2005; Kusaba et al., 2013). Among the endogenous factors that are able to modulate the progression of senescence, it is well established that leaf senescence can be induced, delayed or suppressed by phytohormones (Jibran et al., 2013; Zhang and Zhou, 2013; Khan et al., 2014). Indeed, cytokinins and gibberellins are known to decelerate senescence processes (Gan and Amasino, 1995 for *Nicotiana tabacum*; Yu et al., 2009 for *Paris polyphylla*) while ethylene, JA, ABA and SA are known to promote and accelerate leaf senescence in *Arabidopsis thaliana* (Morris et al., 2000; He et al., 2001; Jing et al., 2005; Zhang and Gan, 2012). Moreover, some altered senescence phenotypes occur after the alteration of phytohormone signaling. For example, AZF2 encodes for a Cys2/His2 type zinc finger protein and its transcript level was up-regulated by ABA, while a loss-of function of AZF2 delayed natural leaf senescence in *A. thaliana* (Li et al., 2012). It was also recently reported that the overexpression of a cytokinin biosynthesis gene (isopentenyltransferase) in transgenic canola (*B. napus* L.) led to a delay in leaf senescence and improved the seed yield under both rainfed and irrigated conditions (Kant et al., 2015).

Finally, when leaf senescence is initiated, several molecular and physiological events occur such as chloroplast breakdown, as well as oxidation and hydrolysis of macromolecules such as lipids, nucleic acids and proteins (Krupinska et al., 2012; Avice and Etienne, 2014; Kim et al., 2016). The breakdown and particularly the hydrolysis of soluble proteins like RuBisCO (ribulose-1,5-biphosphate carboxylase/oxygenase, EC 4.1.1.39) are the most important degradation processes during leaf senescence. In the context of reducing N fertilizers, this recycling of N compounds from source leaves is crucial to satisfy the N demand of growing organs (Demirevska-Kepova et al., 2005; Thoenen et al., 2007; Diaz et al., 2008). It has been demonstrated that the optimization of NRE in oilseed rape is highly related to soluble protein degradation and the improvement of RuBisCO recycling by proteases during leaf senescence (Desclos-Théveniau et al., 2014; Girondé et al., 2015). Protein degradation during senescence is associated with the activity of several protease classes such as APs, MPs, SPs, CPs and the proteasome (Roberts et al., 2012; Diaz-Mendoza et al., 2016). In oilseed rape leaves, N limitation leads to an increase in AP activity during the first phases of senescence [Desclos et al., 2009 (cv. Capitol); Girondé et al., 2016 (cv. Aviso)]. Moreover, Poret et al. (2016) have demonstrated that AP activities remain stable until the late stages of leaf senescence in oilseed rape (cv. Aviso). MPs and the proteasome have also been implicated in leaf senescence. Indeed, proteomic analyses showed that a chloroplastic FtsH and the catalytic β1 subunit of the proteasome were induced in oilseed rape during leaf senescence [Desclos et al., 2009 (cv. Capitol)]. Moreover, the global activity of the proteasome remains stable until the end of leaf senescence in oilseed rape [Poret et al., 2016 (cv. Aviso)]. During leaf senescence, the global activity of SPs is also increased in oilseed rape (cv. Aviso) particularly in response to N limitation, and has been associated with several active SPs such as subtilisins (S8) and POPLs (S9) (Poret et al., 2016). As reported for other species (Bhalerao et al., 2003 for *Populus tremula*; Guo et al., 2004 for *A. thaliana*), the CP class corresponds to the most abundant class of up-regulated proteases during leaf senescence and seems to be crucial for the degradation of soluble proteins in oilseed rape. A recent study showed that some PLCPs and VPEs were present in mature leaves and their activity increased during leaf senescence, particularly under low N conditions [Poret et al., 2016 (cv. Aviso)]. In addition, new CP activities were detected during leaf senescence in oilseed rape, especially under nitrate limitation and they corresponded to the activities of RD21-, SAG12-, XBCP3-, and AALPs [Poret et al., 2016 (cv. Aviso)].

As proteolysis efficiency is essential for the improvement of NRE in leaves of oilseed rape, the genotypic variability of leaf NRE observed by Girondé et al. (2015) in response to N limitation could be linked to contrasted protease activities during leaf senescence. Based on this hypothesis, our first goal was to characterize and compare the senescence-associated protease activities of two genotypes (Ténor and Samouraï) previously identified with contrasting NREs (Girondé et al., 2015). Ténor, unlike Samouraï, is able to maintain its leaf biomass production in response to low N supply and this is essentially due to its comparatively greater degradation of soluble proteins in source leaves (Girondé et al., 2015). Moreover, because senescence is tightly controlled by endogenous phytohormones, the putative genotypic differences in protease activities have been hypothesized as being associated with differential regulation of senescence by the phytohormones. In order to verify this assumption, our second objective was to compare the phytohormone contents between the two genotypes during leaf senescence and to correlate the hormonal patterns with the protease activities.

## Materials and Methods

### Chemicals

Ac-YVAD-cmk, diisopropylfluorophosphate (DFP), E-64 and epoxomicin were provided by SIGMA-ALDRICH^®^. The probes MV201, DCG04, JOPD1, FY01, MVB072, FP-Rh and FP-biotin (Patricelli et al., 2001; Kolodziejek et al., 2011; Richau et al., 2012; Lu et al., 2015) were available in the laboratory. Details are given in Supplementary Table S1 for correspondence between the classes of proteases, their specific inhibitors and activity-based probes.

### Experimental Design

Plants of *B. napus* L. (genotypes Ténor and Samouraï) were cultivated in a greenhouse at the vegetative stage according to previous experiments detailed by Poret et al. (2016). Seedlings were cultivated with 25% Hoagland nutrient solution [1.25 mM Ca(NO_3_)_2_.4H_2_O, 1.25 mM KNO_3_, 0.5 mM MgSO_4,_ 0.25 mM KH_2_PO_4_, 0.2 mM EDTA.2NaFe.3H_2_O, 14 μM H_3_BO_3_, 5 μM MnSO_4_, 3 μM ZnSO_4_, 0.7 μM (NH_4_)_6_Mo_7_O_24_, 0.7 μM CuSO_4_, 0.1 μM CoCl_2_] for 6 weeks. Throughout their growth the plants were subjected to a 16 h photoperiod with a mean temperature of 20°C (day)/15°C (night) and received 400 μmoles photon. s^-1^ m^-2^ of photosynthetically active radiation at the canopy. During this experiment, the N remobilization in source leaves was determined precisely using a pulse-chase ^15^N labeling method. Thus, during the first 6 weeks of culture, plants received nitrate labeled with ^15^N at 2 atom% excess in order to obtain homogenous labeling throughout the plants. After 6 weeks of growth, corresponding to the beginning of treatments [Day 0 (D0)], the ^15^N-nitrate labeling was stopped. Plants were separated into two groups and supplied with 25% Hoagland solution containing two different nitrate concentrations: high (HN: 3.75 mM of CaNO_3_) or low nitrate levels (LN: 0.375 mM CaNO_3_ with compensation for Ca and K elements by adding 1.25 mM CaCl_2_,2H_2_O and 0.875 mM KCl). Leaves were numbered in order of their date of emergence with leaf rank no. 1 (L1) as the first emerged leaf. At D0, i.e., after 6 weeks of culture, leaf rank no. 12 (L12), a mature leaf becoming senescent during the experiment was chosen on the basis of its leaf area determined with a LI-COR 300 area meter (LI-COR, Lincoln, NE, United States) and chlorophyll content measured with a SPAD meter (Soil Plant Analysis Development; Minolta, SPAD-502 model). This leaf rank (L12) has a mean leaf area value of 57.88 cm^2^ ± 1.96% variation and 33.31 cm^2^ ± 6.01% variation for Ténor and Samouraï, respectively; mean SPAD value of 54.16 ± 4.55% variation and 55.33 ± 6.65% variation for Ténor and Samouraï, respectively. During the harvests after 0, 16, and 23 days of treatment (D0, D16, and D23), the chlorophyll and anthocyanin levels of L12 were measured by SPAD and an optical sensor system (Multiplex^®^, Orsay, France; D’Hooghe et al., 2013), respectively. The leaf blade (laminae) of L12 was separated from the petiole and the midrib and was directly frozen at -80°C before further biochemical, proteomic and molecular analyses.

### Quantification of N and ^15^N Contents

The N and ^15^N contents were quantified at D0, D16, and D23 on L12 of Ténor and Samouraï plants subjected to HN or LN supply. The determination was performed with an elemental analyser (EA3000, EuroVector, Milan, Italy) linked to a continuous flow isotope ratio mass spectrometer (IRMS, IsoPrime GV Instruments, Manchester, United Kingdom).

### Profiling of Protease Activities

Soluble proteins were extracted from 200 mg of L12 fresh tissue with 1 mL of water. After centrifugation (5 min, 13,000 *g*, 4°C), concentrations were quantified by protein-dye staining (Bradford, 1976) in equivalent bovine serum albumin (BSA).

The labeling of active proteases was carried out according to Poret et al. (2017). Protein extracts (20 μL; see Supplementary Table S2 for concentrations of soluble proteins) were incubated in a mix containing 0.5 μM of probe (MV201, FY01, or JOPD1), 50 mM of sodium acetate buffer (NaAc, pH 5.5) and 2 mM DTT (Mix final volume: 200 μL). In parallel, 20 μL of protein extract were incubated in a mix of 50 mM Tris-base buffer (pH 7.5) containing 0.5 μM of MVB072 or 0.25 μM of FP-Rh (200 μL final volume). Because probes have not the same affinity for the active site of the targeted hydrolases, the two different mixtures were incubated for 4 h (MV201, FY01, or JOPD1) or 1 h (MVB072 or FP-Rh) under gentle agitation in the dark for better results. As control, equal volumes of soluble protein extracts of L12 (D0, D16, and D23) treated under HN or LN conditions were combined and 20 μL of each mixture were treated as described above. An equal volume of DMSO was added to the No-Probe-Control (NPC) and competition experiments were carried out by performing a pre-treatment for 30 min with 50 μM of E-64 (competition with MV201 and FY01), ac-YVAD-cmk (competition with JOPD1), epoxomicin (competition with MVB072) or DFP (competition with FP-Rh) before adding probes (see Supplementary Table S1 for correspondence between the classes of proteases, their specific inhibitors and activity-based probes). Reactions were stopped by adding 1 mL of ice-cold acetone to precipitate the proteins. After centrifugation (15 min, 16,000 *g*, 4°C), the pellet was dissolved in 2X SDS-PAGE gel-loading buffer (140 mM sodium dodecyl sulfate, 200 mM Tris, 20% glycerol, 5% β-mercaptoethanol, 0.3 mM Bromophenol Blue), heated at 90°C for 10 min and separated on 12% SDS-PAGE gels. The fluorescence of labeled proteins was visualized by scanning the gels using a Typhoon 9400 scanner (GE Healthcare Life Science, excitation wavelength: 532 nm; emission wavelength: 580 nm). Signals were quantified by ImageJ software. To control the protein quantity after electrophoresis, gels were stained with Coomassie Brilliant Blue stain [0.5 g CBB G250, 10% acetic acid, 45% methanol in ultra-pure water (v/v)], destained [10% acetic acid, 40% methanol in ultra-pure water (v/v)] and scanned. Finally, specific protease activity was expressed as fluorescence intensity.mg^-1^ protein (see Supplementary Table S2 for concentrations of soluble proteins).

### Identification of Active Proteases

To identify active proteases previously detected on gels as described above, protein extracts were labeled with biotin-tagged probes and a pull-down of biotinylated proteins was performed with a modified protocol from Poret et al. (2016). Briefly, 900 μg of protein was labeled with 10 μM of DCG04 or FP-biotin in labeling buffer [50 mM sodium acetate buffer (NaAc, pH 5.5), 2 mM DTT for DCG04 or 50 mM Tris-buffer, pH 7.5 for FP-biotin]. Samples were incubated under gentle agitation at room temperature for 4 h (for DCG04) or 1 h (for FP-biotin). An additional aliquot was treated as described above but without probes as the control. As described by Chandrasekar et al. (2014), the reaction was stopped and the biotin-proteins were purified using streptavidin beads. Finally, after separation on 12% SDS-PAGE gels, the eluted proteins were stained using the silver staining procedure described by Blum et al. (1987).

Bands of interest were manually excised and were reduced at 50°C for 1 h with 10 mM dithiothreitol (DTT, GE Healthcare) and alkylated for 1 h in the dark with 55 mM iodoacetamide (IAA, Sigma). The gel fragments were washed several times with water and ammonium carbonate, dehydrated with 0.1% acetonitrile and dried. Trypsin digestion was performed overnight with a dedicated automated system (MultiPROBE II, PerkinElmer). The gel fragments were subsequently incubated twice for 15 min in acetonitrile solution to extract peptides from the gel pieces. Peptide extracts were then dried and dissolved in a buffer containing 3% acetonitrile and 0.1% formic acid for chromatographic elution. Peptides were enriched, separated and analyzed using a 6520 Accurate-Mass Q-TOF LC/MS equipped with an HPLC-chip cube interface (Agilent Technologies, Massy, France). The fragmentation data were interpreted using the Mass Hunter software (version B.03.01, Agilent Technologies). For protein identification, MS/MS peak lists were extracted, converted into mzdata.xml format files and compared with the protein database (NCBInr-*Viridiplantae*) using the MASCOT Daemon search engine (version 2.1.3; Matrix Science, London, United Kingdom). The searches were performed with no fixed modification and with variable modifications for oxidation of methionine, and with a maximum of two missed cleavage sites. MS/MS spectra were searched with a mass tolerance of 20 ppm for precursor ions and 0.6 Da for MS/MS fragments. Only peptides matching an individual ion score > 48 were considered. Proteins with two or more unique peptides matching the protein sequence were considered as a positive identification. The assigned protein of best match is provided alongside the UniProt or NCBI/GenBank accession number. Score, queries matched, peptide matches, different peptide matches, experimental mass and theoretical mass are also presented. In addition, other proteins identified in *B. napus* with the same peptides by MASCOT are presented. Protein sequences were matched against sequences of *Brassicacea* proteins using the NCBI BLAST Protein Database (algorithm blastp) and the best BLAST results are presented with the name of the protein, the organism, the UniProt or NCBI/GenBank accession number and the percentage of sequence identity. Finally, PLCPs were classified according to the classification of Richau et al. (2012) while SPs were classified according to the MEROPS database.

### Immunodetection of SAG12 and RD21

For immunodetection of the CPs SAG12 and RD21, soluble proteins (30 μg) were first denatured with Laemmli 2X buffer (Laemmli, 1970) containing 5% β-mercaptoethanol (v/v) and separated on a 4–15% gradient in SDS-PAGE precast Stain-free gels (Mini-PROTEAN^®^ TGXTM Stain Free, Bio-Rad, Marne-la-Coquette, France). The gels were scanned under UV light with a Gel Doc^TM^ EZ scanner (Bio-Rad^®^, Marne-la-Coquette, France) for detection of proteins and the Western blot to the polyvinylidene difluoride (PVDF) membrane was performed as described by Desclos et al. (2008). The immunodetection of SAG12 was made using specific polyclonal antibody from rabbit provided by Agrisera^®^ [1/2000, diluted in Tris buffer saline – Tween 20 (TBST; Tris 10 mM, NaCl 150 mM, pH8, Tween 20 0.15% (v/v)) containing 5% milk (w/v) to avoid non-specific hybridization]. For immunodetection of RD21, specific polyclonal antibody from rabbit kindly provided by Dr. Hara-Nishimura (Yamada et al., 2001) was used (1/1000, diluted in TBST containing 5% milk). The primary antibody was detected by a secondary antibody from goat coupled with horse-radish peroxidase [(1/10000 and 1/2000 for immunodetection of SAG12 and RD21, respectively) diluted in TBST, Bio-Rad^®^] and detected by enhanced chemiluminescence (ECL kit, Bio-Rad^®^) using a ProXPRESS 2D proteomic Imaging System (PerkinElmer, Courtaboeuf, France).

### Phytohormone Measurements

Phytohormones were extracted by grinding 200 mg of frozen L12 tissue with 1 mL of extraction buffer (methanol 80%, formic acid 1% in ultra-pure water). After 5 min in an ultrasonic bath at 4°C, samples were agitated for 30 min at room temperature and centrifuged at 12,000 *g* (10 min at 4°C). The resulting supernatant containing phytohormones was collected and a second extraction was performed from the resulting pellet. Both supernatants were mixed and evaporated using a SpeedVac. Finally, the pellet was resuspended in 100 μL of methanol containing 1% of formic acid buffer and then analyzed by UPLC-ESI (+/-) –TQD as described in Pan et al. (2010).

Briefly, the extract obtained is analyzed by ultra-high performance liquid chromatography (UPLC) (Acquity of Waters, Guyancourt, France) on a reverse phase column coupled with a double detection in UV-Visible and by mass spectrometry triple quadrupole (Acquity of Waters, Guyancourt, France), which allows a specificity and sensitivity compatible with the quantification of the phytohormones.

### Statistical Analysis

The normality of the data was studied by using the Ryan-Joiner test at 95%. Analysis of variance (ANOVA) and the Newman–Keuls test were performed by using Microsoft^®^ Excel 2010/XLStat^®^ 2014 to compare the means. The non-parametric test of Kruskal–Wallis was carried out if the normality law of the data was not respected. Statistical significance was postulated at *P* < 0.05. Three biological repetitions were analyzed (*n* = 3) for all measurements and all the data are presented as the mean ± standard deviation (SD).

## Results and Discussion

In order to characterize key proteolytic mechanisms capable of distinguishing genotypes with contrasted NRE, our objective was first to identify and compare senescence-associated protease (SAPs) activities of the two genotypes, Ténor (high NRE) and Samouraï (low NRE) (Girondé et al., 2015), and second to associate SAP activities with changes in phytohormonal contents.

### Physiological and Biochemical Changes Associated With Senescence for Ténor vs. Samouraï

The two *B. napus* L. genotypes, Ténor and Samouraï, were subjected to ample (HN: 3.75 mM NO_3_^-^) or low nitrogen supply (LN: 0.375 mM NO_3_^-^) for 23 days after a pulse-chase labeling treatment with ^15^N-NO_3_^-^. Chlorophylls and anthocyanin contents as well as the soluble proteins and the total N and ^15^N contents were followed in a mature leaf (L12, assumed to commence aging and develop as a source tissue during the time course of the experiment) to study senescence progression during the 23 days of the HN or LN treatments (see section “Materials and Methods”).

**FIGURE 1:**
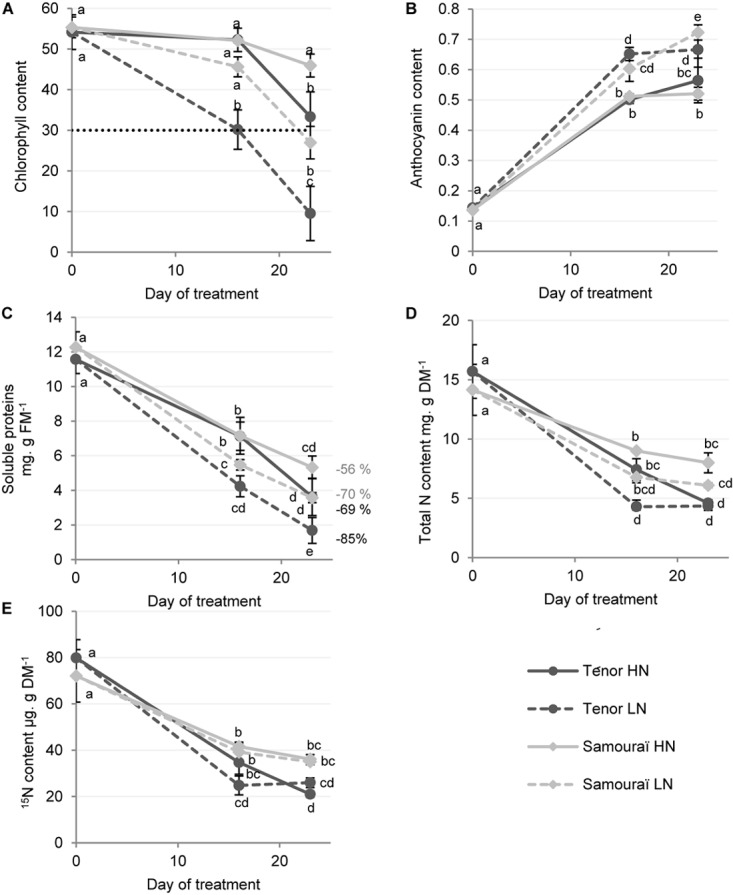
Chlorophyll, anthocyanin, soluble proteins and N and ^15^N contents in leaf blades during senescence in two genotypes of *Brassica napus* L. supplied with high (HN) or low (LN) nitrate for 23 days. Plants (6 weeks old) of two different genotypes (Ténor and Samouraï) were subjected to ample (HN: 3.75 mM NO_3_^-^) or low nitrate supply (LN: 0.375 mM NO_3_^-^) for 23 days. Chlorophyll content of the leaf blades of source leaves (L12) were measured with a SPAD meter **(A)** and were considered as senescent when the chlorophyll content had decreased by at least 40% of the initial value (here the senescence threshold was placed at 30 SPAD units). Anthocyanin content was measured with an optical sensor system (Multiplex) **(B)**. The quantity of soluble proteins was determined after extraction by protein-dye staining **(C)** and the percentages of degradation between D0 and D23 are indicated. The quantity of total nitrogen **(D)** and the quantity of ^15^N **(E)** were measured by isotope-ratio mass spectrometry (IRMS). Vertical bars indicate ± SD of the mean (*n* = 3). Statistical differences are represented by letters (*P* < 0.05, ANOVA, Newman–Keuls test).

As previously shown by Poret et al. (2016) in oilseed rape (cv. Aviso), a limitation of nitrate supply accelerated leaf senescence compared to plants subjected to HN conditions in both genotypes (Figure 1). Indeed, in agreement with the fact that chlorophyll degradation corresponds to one of the first visible processes of aging (Pan et al., 2010), chlorophyll content decreased significantly after 23 days under LN conditions compared to the HN supply and particularly for Ténor (9.5 ± 3.8 SPAD units) compared to Samouraï (26.9 ± 2.3 SPAD units) (Figure 1A). Moreover, the anthocyanin content significantly increased during leaf senescence in the two genotypes and particularly after 23 days under LN supply (Figure 1B). This increase in anthocyanin content during leaf senescence was previously shown in oilseed rape (Poret et al., 2016) and corresponds to a stress symptom in oilseed rape (D’Hooghe et al., 2013). It was recently proposed that the photo-protective function of anthocyanins prevents the risk of photooxidation and enables a tightly controlled and efficient chlorophyll breakdown during leaf senescence (Ougham et al., 2005; Diaz et al., 2006; Koeslin-Findeklee et al., 2015).

As proteolysis efficiency is critical for N recycling performance, the quantity of soluble proteins during senescence was compared in Ténor and Samouraï (Figure 1C). After 23 days, the quantity of soluble proteins was significantly decreased in L12 and this was particularly acute for Ténor under LN conditions with a degradation of 85% of the initial pool of soluble proteins versus only 70% for Samouraï. Then, because the N requirements of growing organs is significantly fulfilled by the transport of N compounds that originate from the recycling of amino acids and soluble proteins in senescing leaves (Demirevska-Kepova et al., 2005; Thoenen et al., 2007), the total N and ^15^N contents were studied (Figure 1D,E). As expected, the amounts of total N and ^15^N strongly decreased during leaf senescence, which could correspond to an increase in N remobilization from the old source leaves to the young sink organs. Moreover, Ténor was characterized by a stronger decrease in the total N and ^15^N contents during the 23 days compared to Samouraï, with a decrease of 70% in ^15^N for Ténor versus only 50% for Samouraï.

In conclusion, Ténor, which is able to maintain its biomass production under N limitation compared to Samouraï (Girondé et al., 2015), is characterized by a stronger decrease in chlorophyll content, an enhanced degradation of soluble proteins under LN conditions and this leads to a stronger decrease in the ^15^N and total N quantities under LN conditions compared to Samouraï.

### Leaf Senescence-Associated Protease (SAP) Activities for Ténor vs. Samouraï

As shown previously, Ténor is able to strongly degrade soluble proteins compared to Samouraï particularly in plants supplied with LN treatment. From these results, two different hypotheses were postulated: (i) the activities of SAPs may be stronger for Ténor than Samouraï and/or (ii) Ténor may present other SAPs activities that could be absent in Samouraï. In order to validate or refute these hypotheses, the aim was to address the typology of protease activities associated with leaf senescence for Ténor and Samouraï. To reach this goal, an appropriate method of labeling active proteases was undertaken at pH 5.5 or 7.5 using activity-based probes specific for different protease classes (van der Hoorn and Kaiser, 2012). The following results allowed validation of the two hypotheses postulated above.

#### Proteasome Activity

**FIGURE 2:**
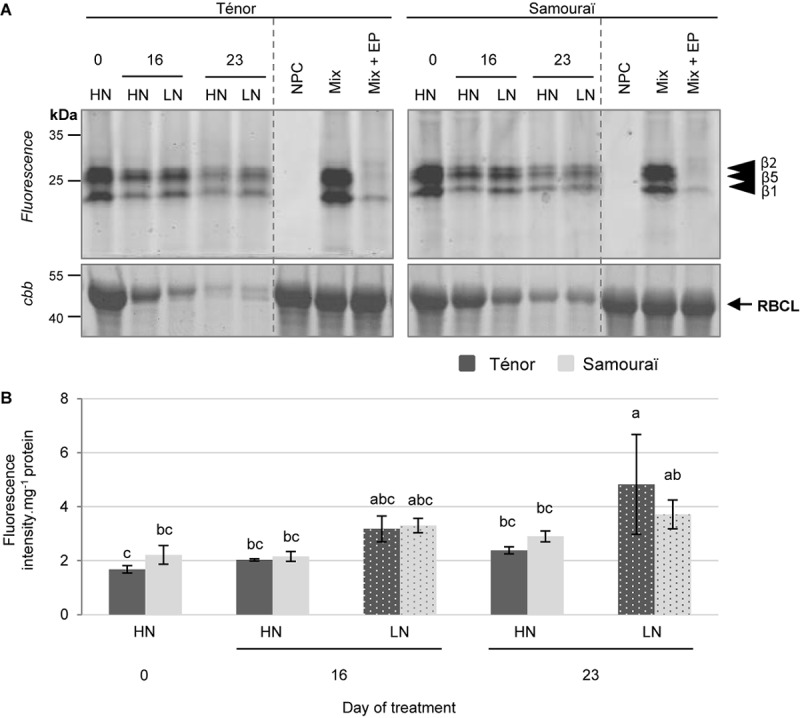
The proteasome activity at pH 7.5 during leaf senescence in two genotypes of *Brassica napus* L. supplied with high (HN) or low (LN) nitrate for 23 days. Soluble proteins were extracted from leaf blade of source leaf (L12) of Ténor or Samouraï plants after 0, 16, and 23 days of HN (3.75 mM NO_3_^-^) or LN (0.375 mM NO_3_^-^) treatment. Samples were subjected to a labeling of protease activity with MVB072 (specific fluorescent probe of the proteasome) (pH 7.5; 1 h labeling). The fluorescence was detected by a scanner after separation of samples by SDS-PAGE **(A)**. Mix corresponds to a mixture of the protein extracts obtained at 0, 16, and 23 days for both treatments (HN and LN) in the presence of MVB072. Mix+EP: mix and epoxomicin (specific inhibitor of the proteasome). NPC, no probe control (absence of MVB072). After incubation, the abundance of RuBisCO large subunit (RBLC) was observed after gel staining by coomassie brilliant blue (*cbb*). In each lane, the total amount of loaded proteins corresponds to 20 μL of protein sample. Positions of catalytic subunits of the proteasome (β1, β2, and β5) are represented by black arrowheads. The proteasome activity characterized by the fluorescence intensity was calculated relative to the protein amount **(B)**. The gel is representative of three biological replicates. Vertical bars indicate ± SD of the mean of three biological replicates. Statistical differences are represented by letters (*P* < 0.05, ANOVA, Newman–Keuls test).

The proteasome is physiologically active under neutral pH therefore activity was studied at pH 7.5. In order to study proteasome activity during senescence, a labeling assay using MVB072 (a specific probe of the proteasome) was carried out (Figure 2, Kolodziejek et al., 2011). The sum of the activities of the three related catalytic subunits (β1, β2, and β5) of the proteasome showed that the activity increased only after 23 days of N limitation in Ténor plants (Figure 2B). These data suggest that the proteasome plays an important role during leaf senescence with an increase in its activity under LN conditions as previously shown in different genotypes of *B. napus* L. during leaf senescence [Desclos et al., 2009 (cv. Capitol); Poret et al., 2016 (cv. Aviso)]. Indeed, Poret et al. (2016) showed that proteasome activity remains stable until the late stages of senescence, whereas Desclos et al. (2009) showed an accumulation of the β1 subunit in response to nitrate limitation or starvation. Nevertheless, this activity is not significantly stronger in Ténor than Samouraï leaves (Figure 2B). These data suggest that the proteasome seems to not be responsible for the enhanced degradation of soluble proteins during the progression of leaf senescence in Ténor compared to Samouraï.

#### Serine Hydrolase (SH) Including Serine Protease (SP) Activities

**FIGURE 3:**
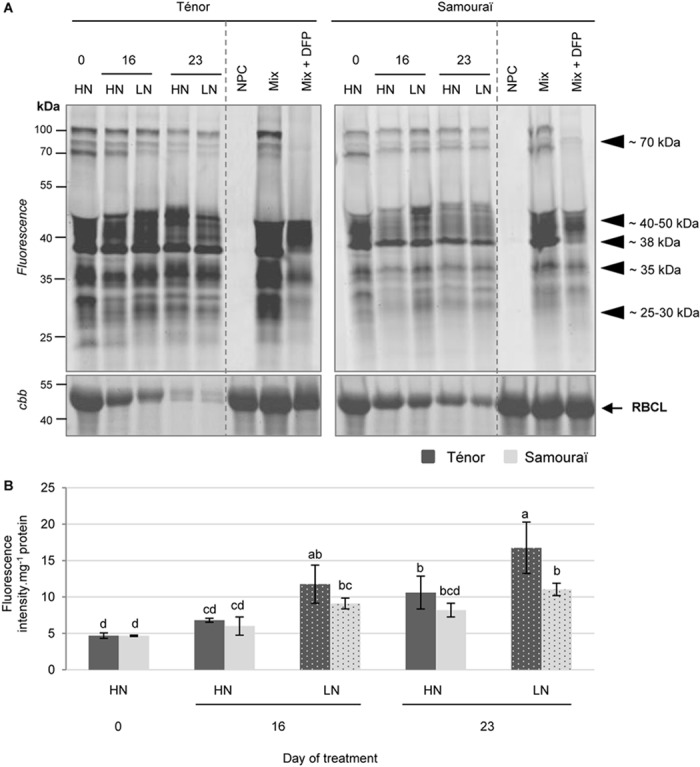
Activity of serine proteases at pH 7.5 during leaf senescence in two genotypes of *Brassica napus* L. supplied with high (HN) or low (LN) nitrate for 23 days. Soluble proteins were extracted from leaf blade of source leaf (L12) of Ténor or Samouraï plants after 0, 16, and 23 days of HN (3.75 mM NO_3_^-^) or LN (0.375 mM NO_3_^-^) treatment. Samples were subjected to a labeling of protease activity with FP-Rh (specific fluorescent probe of serine proteases) (pH 7.5; 1 h labeling). The fluorescence was detected by a scanner after separation of samples by SDS-PAGE **(A)**. Mix corresponds to a mixture of the protein extracts obtained at 0, 16, and 23 days for both treatments (HN and LN) in the presence of FP-Rh. Mix+DFP: mix and diisopropylfluorophosphate (specific inhibitor of serine proteases). NPC: no probe control (absence of FP-Rh). After incubation, the abundance of RuBisCO large subunit (RBLC) was observed after gel staining by coomassie brilliant blue (*cbb*). In each lane, the total amount of loaded proteins corresponds to 20 μL of protein sample. Positions of active proteases are represented by black arrowheads. Serine protease global activity characterized by the fluorescence intensity was calculated relative to the protein amount **(B)**. The gel is representative of three biological replicates. Vertical bars indicate ± SD of the mean of three biological replicates. Statistical differences are represented by letters (*P* < 0.05, ANOVA, Newman–Keuls test).

In order to study SP activities during senescence, labeling with FP-Rh (a specific probe of SHs including SPs) was performed (Figure 3, Patricelli et al., 2001). It was reported that SPs could be located to the vacuole (Parrott et al., 2007) and the chloroplast (Antão and Malcata, 2005), suggesting that SPs could be activated at acidic or neutral pH. In addition, Poret et al. (2016) showed that SH activities at pH 7.5 were strongly activated during leaf senescence in oilseed rape plants. This is why we focused on characterization of the SH activities at pH 7.5. Many of the activities of SPs were detected at 70, 40–50, 38, 35, and 25–30 kDa (Figure 3A) in particular. Quantification of the sum of activities indicated that the global activity of SHs increased during senescence, especially for plants subjected to N limitation for 16 and 23 days for both genotypes (Figure 3B). In addition, the global SH activity was stronger for Ténor than Samouraï plants particularly after 23 days under LN conditions and this could be the result of the stronger activities detected at 38, 35, and 25–30 kDa for Ténor compared to Samouraï (Figure 3B).

To identify SPs responsible for these activities, labeling of active SHs using a biotin-tagged FP probe (Patricelli et al., 2001) was performed followed by purification of the biotinylated proteins (Supplementary Figure S1). This was carried out only for the extract from the senescent leaves of Ténor that was subjected to nitrate limitation over 23 days. Many serine hydrolases were identified at 70, 50, 45, 37, 35, 30, 27, and 25 kDa (Table 1, SHs identifications are detailed in Supplementary Table S3). Some of these hydrolases were identified at different molecular weight cutting zones but we identified 8 different POPLs (S9) at 70 kDa in particular, 2 subtilisins (S8) at 70 kDa, 10 carboxypeptidase-like proteins (SCPLs S10) at 50, 45, 37, 35, 30, and 27 kDa, 5 CXEs at 45, 37, 35, 30, and 27 kDa. In addition, 1 PAE at 45 kDa, 1 Deg protease (S1) at 37 kDa, 3 S-formylglutathione hydrolases at 35 and 30 kDa, 2 thiolesterases at 25 kDa and 6 MESs at 25 kDa were identified after LC MS/MS analyses. The majority of the serine hydrolases have not yet been functionally characterized in *A. thaliana* (van der Hoorn et al., 2011) but they seem to be implicated in various biological processes. Poret et al. (2016) also identified active POPLs during leaf senescence after 23 days of LN conditions in *B. napus* L. (cv. Aviso) at 70 kDa and two of these were also found in leaves of Ténor (BnaA08g30180D [*B. napus*]/A0A078IWH3; BnaC06g11680D [*B. napus*]/CDY06476.1). Moreover, several studies have already shown the role of subtilisin-like protease (S8) during senescence in different species (Roberts et al., 2003, 2006, 2011 for wheat; Parrott et al., 2007 for barley; Poret et al., 2016 for oilseed rape). As reported in wheat by Roberts et al. (2003), this type of serine hydrolase is able to degrade *in vitro* soluble proteins such as RuBisCO. Interestingly, the protease Do-like 1, chloroplastic ([*B. napus*]/XP_013644609.1) was identified at ∼37 kDa. This type of protease is known to play a role in the degradation of protein such as the chloroplastic thylakoid-bound protein D1 of photosystem II (Kato et al., 2015). Finally, many active SHs were identified in the senescing leaves of Ténor after 23 days of nitrate limitation. Many of these active SPs could be responsible for the stronger global activity of SPs in Ténor than in Samouraï. Therefore, additional experiments are required to characterize the function of these different active SHs and SPs between genotypes to determine their role in the proteolysis associated with leaf senescence in oilseed rape.

**Table 1:** Summary of LC-MS/MS identifications of cysteine and serine proteases labeled with the biotin-tagged probes, DCG04 and FP, respectively, in a senescing leaf of *Brassica napus* L. (cv. Ténor) after 23 days of LN treatment.

Protein accession no [*Brassica napus*]/Uniprot or NCBI accession no.	Classification	Putative location
***Cysteine proteases (PLCPs)***		
BnaA10g05390D [*Brassica napus*]/CDY06760	RD21-like	Vac
BnaA08g04080D [*Brassica napus*]/A0A078FVG4	RD21-like	Vac
Cysteine proteinase RD21a [*Brassica napus*]/XP_013718810	RD21-like	Vac
BnaA06g36920D [*Brassica napus*]/A0A078G7A3	RD21-like	Vac
Cysteine proteinase RD19a-like [*Brassica napus*]/XP_013701923.1	RD19-like	Vac
Cysteine proteinase RD19a [*Brassica napus*]/XP_013747708.1	RD19-like	Vac
Senescence-specific cysteine protease [*Brassica napus*]/Q9SQH3	SAG12-like	Vac
BnaA06g40240D [*Brassica napus*]/A0A078J304	SAG12-like	Vac
Low-temperature-induced cysteine proteinase-like isoform X2 [*Brassica napus*]/XP_013696759.1	XBCP3-like	Vac
BnaA06g05780D [*Brassica napus*]/CDX93470.1	XBCP3-like	Vac
BnaCnng01440D [*Brassica napus*]/CDY07129.1	Cathepsin-B	Vac
BnaA09g52180D [*Brassica napus*]/A0A078J5J7	Cathepsin-B	Vac
BnaC09g35690D [*Brassica napus*]/CDX80173.1	AALP-like	Vac
BnaC01g26060D [*Brassica napus*]/CDX68528.1	Zingipain	Vac
**Serine proteases SPs**		
Subtilisin-like protease SBT1.7 [*Brassica napus*]/XP_013654072.1	Subtilisins S8	EC/Vac
BnaCnng41800D [*Brassica napus*]/A0A078JDZ2	Subtilisins S8	EC/Vac
Acylamino-acid-releasing enzyme-like isoform X1 [*Brassica napus*]/XP_013655680.1	POPLs S9	?
BnaA08g30180D [*Brassica napus*]/A0A078IWH3	POPLs S9	?
BnaCnng64630D [*Brassica napus*]/A0A078JVT3	POPLs S9	?
BnaCnng28400D [*Brassica napus*]/A0A078J055	POPLs S9	?
Prolyl endopeptidase-like [*Brassica napus*]/XP_013656097.1	POPLs S9	?
BnaC06g11680D [*Brassica napus*]/CDY06476.1	POPLs S9	?
Acylamino-acid-releasing enzyme-like [*Brassica napus*]/XP_013644416.1	POPLs S9	?
Prolyl endopeptidase-like [*Brassica napus*]/XP_013642253.1	POPLs S9	?
BnaA06g18620D [*Brassica napus*]/CDX99171.1	SCPLs S10	Vac
BnaC01g38630D [*Brassica napus*]/A0A078J0P9	SCPLs S10	Vac
Serine carboxypeptidase-like 35 [*Brassica napus*]/A0A078G963	SCPLs S10	Vac
BnaA04g07190D [*Brassica napus*]/A0A078HQ25	SCPLs S10	Vac
BnaA01g06330D [*Brassica napus*]/A0A078GRW5	SCPLs S10	Vac
Serine carboxypeptidase-like 29 [*Brassica napus*]/A0A078IYZ5	SCPLs S10	Vac
BnaA08g12880D [*Brassica napus*]/A0A078GF58	SCPLs S10	Vac
Lysosomal Pro-X carboxypeptidase-like [*Brassica napus*]/XP_013643207.1	SCPLs S10	Vac
BnaA04g16130D [*Brassica napus*]/A0A078GVN3	SCPLs S10	Vac
BnaA10g23100D [*Brassica napus*]/CDX69961.1	SCPLs S10	Vac
Protease Do-like 1, chloroplastic [*Brassica napus*]/XP_013644609.1	Deg SPs	Chl


*Chl, chloroplast; EC, extracellular; Vac, vacuolar compartments. The assigned protein of best match is provided alongside the UniProt or NCBI/GenBank accession number and proteases were classified according to Richau et al. (2012) for CPs or the MEROPS database for SPs. Putative cellular locations of these proteases are given based on the data reported by Roberts et al. (2012) and Diaz-Mendoza et al. (2016)*.

#### Cysteine Proteases (CPs): PLCPs and VPEs

**FIGURE 4:**
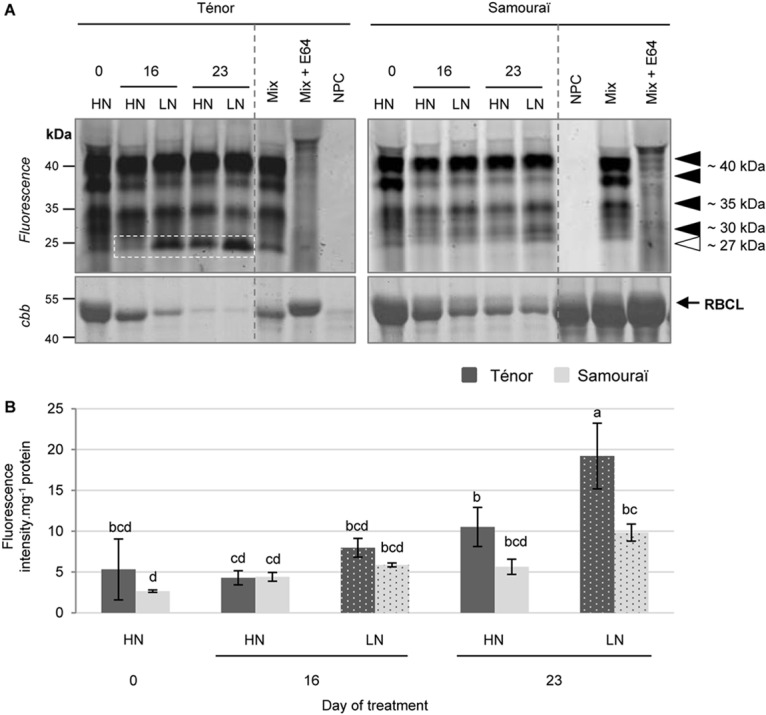
Detection of PLCP activities at pH 5.5 during leaf senescence in two genotypes of *Brassica napus* L. supplied with high (HN) or low (LN) nitrate for 23 days. Soluble proteins were extracted from leaf blade of source leaf (L12) of Ténor or Samouraï plants after 0, 16, and 23 days of HN (3.75 mM NO_3_^-^) or LN (0.375 mM NO_3_^-^) treatment. Samples were subjected to a labeling of protease activity with MV201 (specific fluorescent probe of PLCPs) (pH 5.5; 4 h labeling). The fluorescence was detected by a scanner after separation of samples by SDS-PAGE **(A)**. Mix corresponds to a mixture of the protein extracts obtained at 0, 16, and 23 days for both treatments (HN and LN) in the presence of MV201. Mix+E64: mix and E64 (specific inhibitor of cysteine proteases). NPC, no probe control (absence of MV201). After incubation, the abundance of RuBisCO large subunit (RBLC) was observed after gel staining by coomassie brilliant blue (*cbb*). In each lane, the total amount of loaded proteins corresponds to 20 μL of protein sample. Positions of active proteases are represented by black arrowheads while the white arrowhead shows senescence-induced proteases. PLCP global activity characterized by the fluorescence intensity was calculated relative to the protein amount **(B)**. The gel is representative of three biological replicates. Vertical bars indicate ± SD of the mean of three biological replicates. Statistical differences are represented by letters (*P* < 0.05, ANOVA, Newman–Keuls test).

**FIGURE 5:**
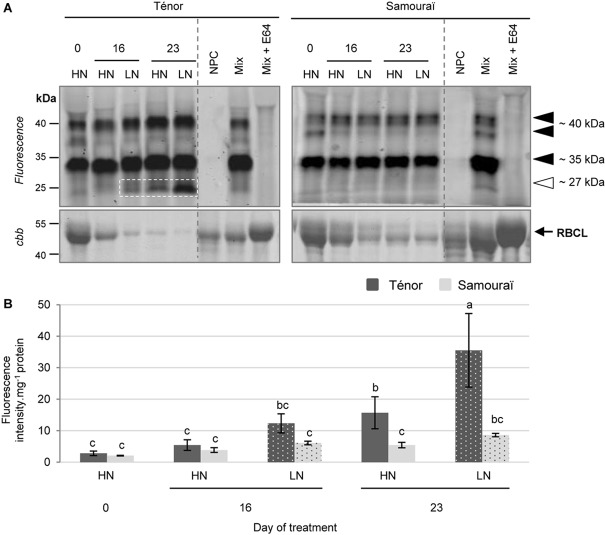
Detection of aleurain-like proteases (AALP) and other PLCP activities at pH 5.5 during leaf senescence in two genotypes of *Brassica napus* L. supplied with high (HN) or low (LN) nitrate for 23 days. Soluble proteins were extracted from leaf blade of source leaf (L12) of Ténor or Samouraï plants after 0, 16, and 23 days of HN (3.75 mM NO_3_^-^) or LN (0.375 mM NO_3_^-^) treatment. Samples were subjected to a labeling of protease activity with FY01 (specific fluorescent probe of AALPs and other PLCPs) (pH 5.5; 4 h labeling). The fluorescence was detected by a scanner after separation of samples by SDS-PAGE **(A)**. Mix corresponds to a mixture of the protein extracts obtained at 0, 16, and 23 days for both treatments (HN and LN) in the presence of FY01. Mix+E64: mix and E64 (specific inhibitor of cysteine proteases). NPC: no probe control (absence of FY01). After incubation, the abundance of RuBisCO large subunit (RBLC) was observed after gel staining by coomassie brilliant blue (*cbb*). In each lane, the total amount of loaded proteins corresponds to 20 μL of protein sample. Positions of active proteases are represented by black arrowheads, while the white arrowhead shows senescence-induced proteases. AALP global activity, characterized by the fluorescence intensity, was calculated relative to the protein amount **(B)**. The gel is representative of three biological replicates. Vertical bars indicate ± SD of the mean of three biological replicates. Statistical differences are represented by letters (*P* < 0.05, ANOVA, Newman–Keuls test).

**FIGURE 6:**
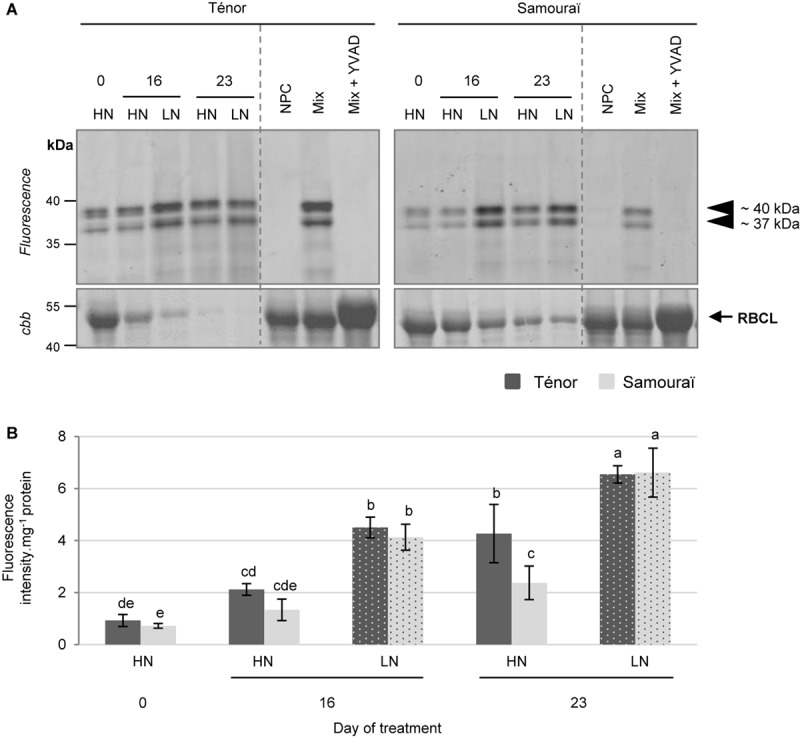
Vacuolar processing enzyme activities at pH 5.5 during leaf senescence in two genotypes of *Brassica napus* L. supplied with high (HN) or low (LN) nitrate for 23 days. Soluble proteins were extracted from leaf blade of source leaf (L12) of Ténor or Samouraï plants after 0, 16, and 23 days of HN (3.75 mM NO_3_^-^) or LN (0.375 mM NO_3_^-^) treatment. Samples were subjected to a labeling of protease activity with JOPD1 (specific fluorescent probe of VPEs) (pH 5.5; 4 h labeling). The fluorescence was detected by a scanner after separation of samples by SDS-PAGE **(A)**. Mix corresponds to a mixture of the protein extracts obtained at 0, 16, and 23 days for both treatments (HN and LN) in the presence of JOPD1. Mix+YVAD: mix and YVAD (specific inhibitor of VPEs). NPC, no probe control (absence of JOPD1). After incubation, the abundance of RuBisCO large subunit (RBLC) was observed after gel staining by coomassie brilliant blue (*cbb*). In each lane, the total amount of loaded proteins corresponds to 20 μL of protein sample. Positions of active proteases are represented by black arrowheads. VPE global activity, characterized by the fluorescence intensity, was calculated relative to the protein amount **(B)**. The gel is representative of three biological replicates. Vertical bars indicate ± SD of the mean of three biological replicates. Statistical differences are represented by letters (*P* < 0.05, ANOVA, Newman–Keuls test).

Because CPs are located in the vacuole or in senescence- associated vesicles (SAVs) (Otegui et al., 2005; Martínez et al., 2007; Kato et al., 2015; Otegui, 2018), the labeling of CP activities was performed under acidic pH (5.5) according to Poret et al. (2016). The protease activity profiling was undertaken with the fluorescent probes MV201 (specific to papain-like cysteine proteases, PLCPs), FY01 (specific to AALPs and PLCPs) and JOPD1 (a probe specific for vacuolar processing enzymes, VPEs) (Figure 4–6).

Many activities of PLCPs were detected using MV201 (Figure 4A) particularly at ∼40, ∼35, ∼30, and ∼27 kDa. The sum of the PLCP activities was quantified (Figure 4B) and the global activity significantly increased during senescence in leaves of plants subjected to the LN supply during the 23 days. Otherwise, the global PLCP activity was stronger for Ténor than Samouraï after 23 days of LN treatment. This strong PLCP activity was related to the increase in protease activities at ∼40 kDa and particularly due to the appearance of a new activity at ∼27 kDa for Ténor but not for Samouraï (Figure 4A).

Moreover, several AALP and PLCP activities were detected using FY01 (Figure 5A), particularly at ∼40, ∼35, and ∼27 kDa. The quantification of the sum of these activities (Figure 5B) showed that the global activity of the aleurain-like and PLCP proteases was significantly increased during senescence in leaves of Ténor alone in response to a nitrate limitation of 23 days. The AALP and PLCP protease activities were increased particularly at ∼40 and ∼35 kDa while a strong new activity was detected at ∼27 kDa. Because the AALPs are a sub-group of the PLCPs, this strong activity at ∼27 kDa seems to contribute to the new activity for PLCPs at this molecular weight (Figure 4A).

Finally, 2 VPE activities were detected after labeling with JOPD1 (Figure 6A) at ∼40 and ∼37 kDa. The sum of these activities was quantified (Figure 6B) and the global activity of the VPEs increased during senescence particularly in plants subjected to an N limitation, regardless of the genotype. In contrast to the PLCPs and AALPs, VPEs do not seem to be responsible for the contrasted proteolytic processes between Ténor and Samouraï observed in Figure 1C.

To identify the PLCPs and AALPs responsible for the increased total CP activity for Ténor compared to Samouraï, labeling with the biotin-tagged DCG04 probe was performed followed by a pull-down of biotinylated proteins (Supplementary Figure S1). As for SPs, this was only performed on the extract from senescent leaves of Ténor subjected to LN supply after 23 days of treatment. Many PLCPs were identified at ∼40, ∼35, ∼30, ∼27, and ∼25 kDa (Table 1, see detailed data on protein identifications in Supplementary Table S4). As for the SPs, some of these PLCPs were identified in different molecular weight zones but we identified 5 different RD21-like proteases, 2 RD19-like proteases, 2 SAG12-like proteases, 2 Cathepsin-B, 2 XBCP3-like proteases, 1 AALP and 1 zingipain.

The CPs are the most up-regulated proteases during leaf senescence [Bhalerao et al. (2003) in *Populus tremula*] and CP activities can represent 44% of the total protease activity in senescent tissues of *B. oleracea* (Coupe et al., 2003). This class of proteases is involved in proteolytic processes during senescence in wheat (Thoenen et al., 2007) and is clearly associated with RuBisCO degradation during sequential leaf senescence in the vegetative stages of *B. napus* L. (Poret et al., 2016). Interestingly, many PLCPs are involved in proteolytic processes during senescence in *A. thaliana*, such as some of the RD21-like proteases, SAG12-like proteases, AALPs, Cathepsin-B proteases or RD19-like proteases (Guo et al., 2004; Otegui et al., 2005; Richau et al., 2012; Ge et al., 2016; James et al., 2018; Otegui, 2018). A recent study also showed that activities of some RD21-like, SAG12-like, and XBCP3-like proteases as well as AALP were especially increased during leaf senescence in *B. napus* L. plants submitted to N limitation [Poret et al., 2016 (cv. Aviso)]. Moreover, BnaA10g05390D [*B. napus*]/CDY06760 (RD21-like protease) and BnaA06g05780D [*B. napus*]/CDX93470.1 (XBCP3-like protease) were also identified as proteases involved in the proteolytic process in the genotype Aviso by Poret et al. (2016).

The main goal of this experiment was to identify proteases responsible for the strong activity that appeared at ∼27 kDa in Ténor but not in Samouraï for PLCPs and AALPs (Figure 4A,5A) and which could be associated with the difference in proteolytic performance between the two genotypes. Many proteases were identified at ∼27 and ∼25 kDa (Table 1 and Supplementary Table S4) as 3 different RD21-like proteases, 2 cathepsin-B proteases, 2 SAG12-like proteases, 1 RD19-like protease, 2 XBCP3-like proteases and 1 AALP, which could have been responsible for the strong activity at this molecular weight observed with FY01 labeling (Figure 5A). None of these proteases were strongly active during leaf senescence in Samouraï after 23 days of LN treatment compared to Ténor. This is why some of these activities might be associated with the difference in proteolytic performance between the two genotypes.

**FIGURE 7:**
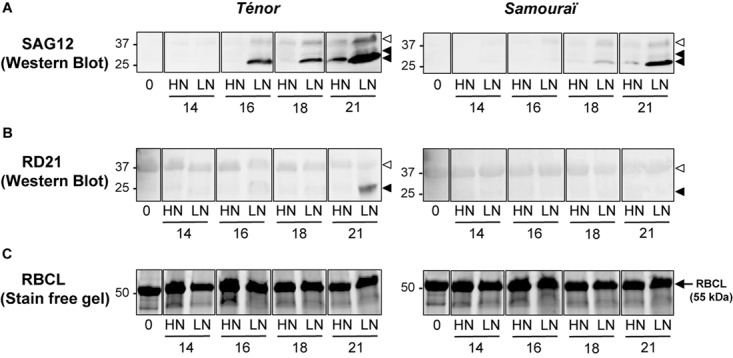
Immunodetection of SAG12 **(A)**, and RD21 **(B)** during leaf senescence in two genotypes of *Brassica napus* L. supplied with high (HN) or low (LN) nitrate during 21 days. Soluble proteins were extracted from leaf blade of source leaf (L12) of Ténor or Samouraï plants after 0, 16, and 23 days of HN (3.75 mM NO_3_^-^) or LN (0.375 mM NO_3_^-^) treatment. Thirty μg of soluble proteins per lane were separated on a 4–15% gradient in SDS-PAGE precast Stain-free gels (Bio-Rad) and gels were scanned under UV light to show the total amount of input proteins (as observed for the large subunit of RuBisCO (RBCL) presented in **(C)**. After the transfer to the polyvinylidene difluoride (PVDF) membrane, the immunodetection was realized with an antibody specific of proteins SAG12 and RD21A. White head arrow: immature form, black head arrow: mature form. The gel and Western Blots are representative of three biological replicates (for details see section “Materials and Methods”).

The *SAG12* gene encodes for a CP and is considered as a senescence-associated gene marker (Lohman et al., 1994). Desclos et al. (2009) have shown an up-regulation of *SAG12* expression and SAG12 protein abundance in senescent leaves of oilseed rape (cv. Capitol) subjected to nitrate limitation. The activity of this type of protease has also been demonstrated in senescing leaves of genotype Aviso subjected to nitrate limitation (Poret et al., 2016). This genotype is able to maintain its leaf biomass production in response to low N supply (Girondé et al., 2015) in a similar way to Ténor. In order to verify that the difference of SAG12 and RD21 activities between both genotypes are related with the abundance of their active forms, the immunodetection of SAG12 and RD21 was carried out in senescent leaves of Ténor or Samouraï plants after 0, 14, 16, 18, and 21 days of HN or LN treatment (Figure 7). Compared to Samouraï, the results clearly showed that the abundance of mature form of SAG12 (26–27 kDa) was greater than immature form (40 kDa) in senescing leaves of Ténor plants after 16 days of N limitation or 21 days under HN conditions (Figure 7A). In both genotypes, the immature form of RD21 (37 kDa, Figure 7B) was detected after 14 days of HN or LN conditions but the abundance is very low. Interestingly, the abundance strongly increased in response of 21 days of N limitation only in senescing leaves of Ténor (Figure 7B). These western blot results confirmed that the active forms of RD21 and SAG12 are more abundant in Ténor and are in agreement with the stronger activity of these CPs in Ténor than in Samouraï leaves observed after 23 days under LN conditions (Figure 4).

In conclusion, the strong proteolytic performances of Ténor during the leaf senescence associated with N limitation (Figure 1C) seem to be closely linked to stronger SP and CP activities, and induction of specific CP activities (SAG12 and RD21) (Figure 3–7 and Table 1) than in Samouraï.

### Phytohormone Contents During Leaf Senescence in Ténor vs. Samouraï

**FIGURE 8:**
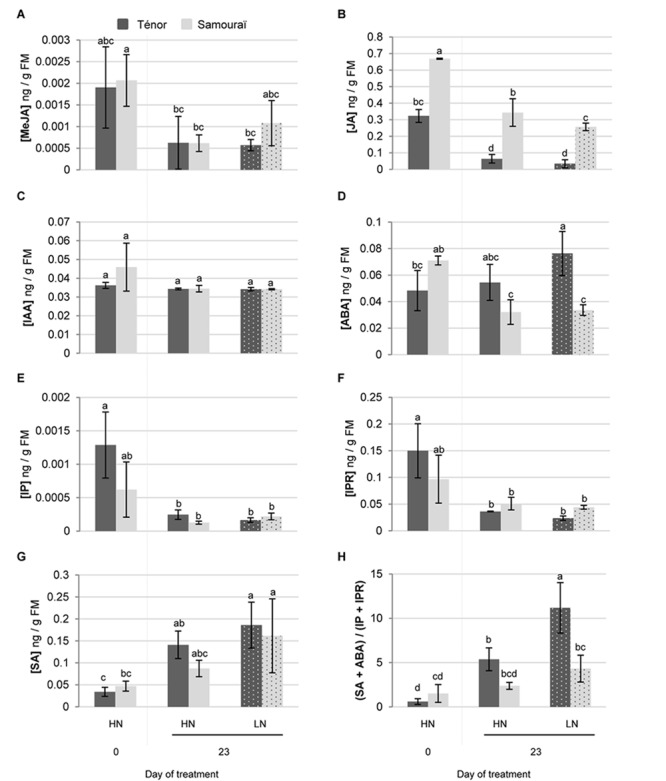
Changes to phytohormone contents during leaf senescence of two genotypes of *Brassica napus* L. supplied with high (HN) or low (LN) nitrate for 23 days. Phytohormones (**A**: MeJA, methyl jasmonate; **B**: JA, jasmonic acid; **C**: IAA, indole acetic acid; **D**: ABA, abscisic acid; **E**: IP, isopentenyladenine; **F**: IPR, isopentenyladenosine and **G**: SA, salicylic acid) were extracted in a methanol/formic acid buffer after 0 and 23 days of HN (3.75 mM NO_3_^-^) or LN (0.375 mM NO_3_^-^) for two genotypes of *Brassica napus* L. (Ténor and Samouraï) and measured by UPLC-MS (for details see section “Materials and Methods”). The ratio of phytohormone contents (SA+ABA)/(IP+IPR) was calculated at D0 and D23 **(H)**. Vertical bars indicate ± SD of the mean of three biological replicates. Statistical differences are represented by letters (*P* < 0.05, ANOVA, Newman–Keuls test).

The senescence process is highly regulated by different endogenous factors that include the phytohormones (Jibran et al., 2013; Khan et al., 2014). Therefore, the difference in leaf-senescence protease activities between Ténor and Samouraï could be associated with differences in the regulation of senescence by phytohormones. To validate or refute this hypothesis, our goal was to study and compare phytohormone contents between Ténor and Samouraï during leaf senescence and correlate the hormonal patterns with the protease activities. To reach this goal, several phytohormone contents were measured by UPLC-ESI (+/-) -TQD in plants of Ténor and Samouraï subjected to HN or LN supply for 23 days (Figure 8).

Surprisingly, the MeJA content decreased during leaf senescence regardless of the treatment and the genotype (Figure 8A) whereas it has been demonstrated that exogenous application of MeJA up-regulates the transcript abundance of genes that are markers of developmental senescence in *A. thaliana* (Jung et al., 2007). Similarly, the JA content decreased during leaf senescence in *B. napus* L. regardless of the nitrate supply or the genotype (Figure 8B) while JA biosynthesis is upregulated in senescing leaves of *A. thaliana* (Seltmann et al., 2010). Otherwise, the auxin (IAA) content remained stable during leaf senescence irrespective of the nitrate treatment and the genotype (Figure 8C). The role of auxin (IAA) in senescence is not clear. Indeed, it has been demonstrated that the IAA-induced gene, *SAUR36*, was up-regulated during senescence while its dysfunction induced a delay to senescence in *A. thaliana* (Hou et al., 2013). Contrastingly, a senescence repressor role for IAA was demonstrated in *A. thaliana* with the down-regulation of several senescence-associated genes (SAGs), including *SAG12*, by an exogenous application of IAA (Noh and Amasino, 1999; Kim et al., 2011). MeJA, JA and IAA do not seem to be associated with the different behaviors observed during leaf senescence in Ténor and Samouraï because the levels were quite similar, and therefore their roles in senescence processes remain unclear in *B. napus* L.

Compared to the initial level (Day 0), the ABA content significantly increased during leaf senescence in Tenor after 23 days under nitrate limitation, while it decreased in Samouraï (Figure 8D). A promotive role of ABA has already been demonstrated during senescence in *A. thaliana* with the up-regulation of *SAG113* (a senescence associated gene) linked with an increase in the ABA endogenous content (Zhang et al., 2012). Moreover, endogenous ABA content increases during senescence in many plant species such as *Avena sativa* (Lim et al., 2007) and *A. thaliana* (Zhang et al., 2012).

The SA content increased during leaf senescence in plants subjected to N limitation for 23 days, regardless of the genotype (Figure 8G). This is correlated with the fourfold increase in SA levels reported in senescent leaves of *A. thaliana* by Morris et al. (2000). Moreover, these authors have also reported that a dysfunction of SA synthesis in *A. thaliana* leads to a delay in senescence and a down-regulation of *SAG12* expression (Morris et al., 2000).

The contents of two intermediate molecules of cytokinin synthesis, IP and isopentenyladenosine (IPR), decreased during senescence in plants regardless of the N treatment and the genotype (Figure 8E,F). This is correlated with the general decrease in cytokinin content observed during leaf senescence in *N. tabacum* while an increase in the endogenous cytokinin level leads to a delay in senescence processes (Yu et al., 2009). Our results are also in agreement with the fact that over-expression of the isopentenyltransferase gene, responsible for cytokinin biosynthesis, generally leads to senescence delay and stay-green phenotypes in numerous species including *B. napus* (Kant et al., 2015; Zeng and Zhao, 2016). Because ABA, SA and cytokinins seem to be strongly involved in the regulation of leaf senescence in *B. napus* L., we focused our analysis on the content of these phytohormones. The change in the ratio between phytohormones that accelerate leaf senescence, such as ABA and SA, and those that negatively regulate senescence, such as cytokinins was analyzed (Figure 8H). The ratio (SA + ABA)/(IP + IPR) increased greatly during leaf senescence in Ténor plants after 23 days of treatment, and particularly after N limitation, whereas it remained stable for Samouraï leaves regardless of the nitrate treatment. This ratio allowed the two genotypes to be distinguished during senescence as well as a clear separation between the N treatments in Ténor during senescence. This ratio was also clearly correlated with the activities of the PLCPs, the AALP and the SPs during senescence with Pearson correlation coefficients of 0.866, 0.932, and 0.905, respectively (*p*-value < 0.0001, data not shown). These data suggested that different hormonal balances during senescence between Ténor and Samouraï could be associated with stronger PLCP and SP activities and proteolytic performance in Ténor compared to Samouraï during senescence, particularly under N limitation. As already described, phytohormones like ABA and SA are able to up-regulate protease transcripts or protease abundance and dysfunction in SA synthesis during senescence leads to down-regulation of *SAG12* gene expression in *A. thaliana* (Morris et al., 2000). Further, ABA has been shown to enhance RuBisCO degradation in detached leaves of *Oryza sativa* L. floated on a solution containing ABA compared to those on a solution without ABA (Fukayama et al., 2010). This was correlated with an increase in SDS-dependent protease activities (with an optimum pH 5.5 suggesting a vacuolar localization) that significantly increased with ABA treatment. These types of protease activities are also increased during leaf senescence in rice (Fukayama et al., 2010).

In addition to these data, a preliminary study that infiltrated exogenous SA or water in mature leaves of oilseed rape (cv. Aviso) was performed (Supplementary Figure S2). The degradation of RuBisCO was followed *in vitro* in the presence or absence of MG132 (an inhibitor of CPs and the proteasome) in these leaves. When leaf tissues were infiltrated with SA, the level of RuBisCO degradation was strongly reduced in the presence of MG132 (with a rate of degradation of 32% versus 60% in the absence of MG132) compared to water infiltration (rate of degradation of 46% versus 59% in the absence of MG132) (Supplementary Figure S2). This enhanced inhibition of RuBisCO degradation by MG132 after SA infiltration (inhibition of 46% versus 22% for water infiltration, Supplementary Figure S2) suggests that the contribution of CPs and the proteasome activities to proteolysis is more important after SA application and reinforces the assumption that SA is involved in the regulation of CP and proteasome activities in leaves of oilseed rape. According to these results observed during leaf senescence, we have recently demonstrated that the infiltration of ABA and SA in cotyledons of oilseed rape (Ténor) provokes the induction of senescence and several cysteine and SP activities in cotyledons (Poret et al., 2017). Nevertheless, additional experiments will be required to validate the postulate that these phytohormone patterns could regulate PLCP or SP protease activities.

## Conclusion

The genotype Ténor, which unlike Samouraï is temporarily able to maintain its biomass production in response to nitrate limitation (Girondé et al., 2015), demonstrates enhanced senescence processes in response to N restriction including a greater decrease in chlorophyll content, efficient degradation of soluble proteins and a greater decrease in the amount of N in senescing leaves. This efficient proteolytic performance is associated with (i) strong increases in SP and PLCP activities and (ii) the appearance of new PLCP activities such as RD21 or SAG12, which is absent or very low in Samouraï leaves during senescence. Moreover, in contrast to Samouraï, Ténor is characterized by a hormonal balance (SA + ABA)/(IP + IPR) that promotes senescence. This high ratio between phytohormones positively regulating senescence and phytohormones negatively regulating senescence may be correlated with more efficient protein degradation *via* the induction of PLCP/SP activities for Ténor compared to Samouraï during senescence, particularly under nitrate limitation. More specific investigations will be necessary to validate the phytohormone regulation of protease activities during senescence.

## Author Contributions

MP and J-CA contributed to the experimental design and tissue sampling. MP carried out the protease activity profiling using activity-dependent fluorescent probes with the help of BC. MP, SD, T-HK, and B-RL carried out the phytohormones extractions, measurements, and chromatograms analyses. MP, FM, IH-N, and J-CA performed other biochemical measurements, proteases analyses, Western Blots, statistical analyses, interpretation of data, and drafting the article. MP, RvdH, AB, T-HK, B-RL, and J-CA were involved in revising the manuscript for important intellectual content.

## Conflict of Interest Statement

The authors declare that the research was conducted in the absence of any commercial or financial relationships that could be construed as a potential conflict of interest.

## Abbreviations

AALP: aleurain-like protease
ABA: abscisic acid
AP: aspartic protease
CP: cysteine protease
CXE: carboxylesterase
HN: high nitrate
IAA: indole acetic acid, auxin
IP: isopentenyladenine
JA: jasmonic acid
LN: low nitrate
MeJA: methyl jasmonate
MES: methylesterase
MP: metalloprotease
N: nitrogen
NPC: no probe control
NRE: nitrogen remobilization efficiency
NUE: nitrogen use efficiency
PAE: pectinacetylesterase
PLCP: papain-like cysteine protease
POPL: prolyloligopeptidase-like protease
RBCL: large subunit of RuBisCO
RuBisCO: ribulose-1,5-biphosphate carboxylase/oxygenase
SA: salicylic acid
SAP: senescence associated protease
SCPL: serine carboxypeptidase-like protein
SP: serine protease
VPE: vacuolar processing enzyme
