# Supplementary material for: A Genotypic Comparison Reveals That the Improvement in Nitrogen Remobilization Efficiency in Oilseed Rape Leaves Is Related to Specific Patterns of Senescence-Associated Protease Activities and Phytohormones
Source: Front Plant Sci. 2019 Feb 4;10:46. doi: 10.3389/fpls.2019.00046 (PMC6369165; doi:10.3389/fpls.2019.00046)
Supplement: Supplementary file 1 [file Data_Sheet_1.PDF]

Table S1 I Different classes of proteases studied in this work, their specific inhibitors and activity-based probes.

Class of protease	Sub-family	Inhibitor	Probe with fluorescent tag	Probe with biotin tag
Cys proteases	PLCPs (C1)	E-64	MV201	DCG04
	Aleurain-like protéases	E-64	FY01	-
	VPE (C14)	Ac-YVAD-cmk	JOPD1	-
Ser proteases	Subtilisins (S8), POPLs (S9), SCPLs (S10)	DFP	FP-Rh	FP-biotin
Proteasome	T3	Epoxomicin	MVB072	-

Table S2 I Soluble proteins concentrations in leaf blades during senescence of two genotypes of *Brassica napus* L. supplied with high (HN) or low (LN) nitrate for 23 days.
Plants (6 weeks old) of two different genotypes (Ténor and Samouraï) were subjected to ample (HN: 3.75 mM NO_3^-) or low nitrate supply (LN: 0.375 mM NO_3^-) for 23 days. The concentration of soluble proteins was determined after extraction by protein-dye staining. Data indicate ± SD of the mean (*n=3*).

**Soluble protein concentrations (µg/µL)**			
Day of treatment	Treatment	Ténor	Samouraï
D0	HN	4.77 ± 0.41	4.94 ± 0.38
D16	HN	2.91 ± 0.31	2.91 ± 0.38
D16	LN	1.71 ± 0.24	2.22 ± 0.1
D23	HN	1.50 ± 0.43	2.16 ± 0.28
D23	LN	0.68 ± 0.31	1.46 ± 0.1

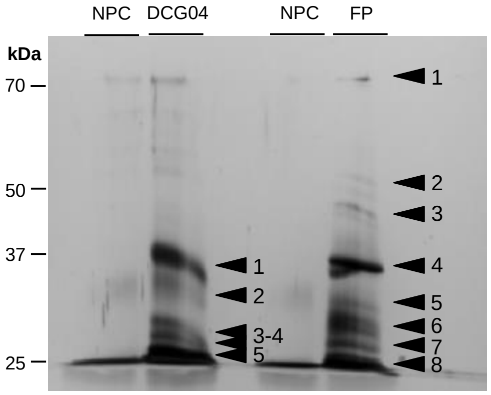

Figure S1 I Detection of PLCPs and SPs labelled with DCG04 and FP-biotin in a senescing leaf of *Brassica napus* L. (cv. Ténor) after 23 days of LN treatment

To characterize PLCPs and SPs observed on the figures 3 and 4, a labeliing with the probes DCG04 and FP-biotin carrying a biotin tag was carried out using a pull-down of biotinylated proteins. This was performed only for the extract from the senescent leaf of the genotype Ténor subjected to LN supply after 23 days. The biotin-labelling reaction was stopped and the proteins were purified using streptavidin beads. After SDS-PAGE, proteins were detected by staining with silver nitrate (for details see "Materials and Methods"). NPC corresponds to the no probe controls. Black arrowheads correspond to the excised zones.

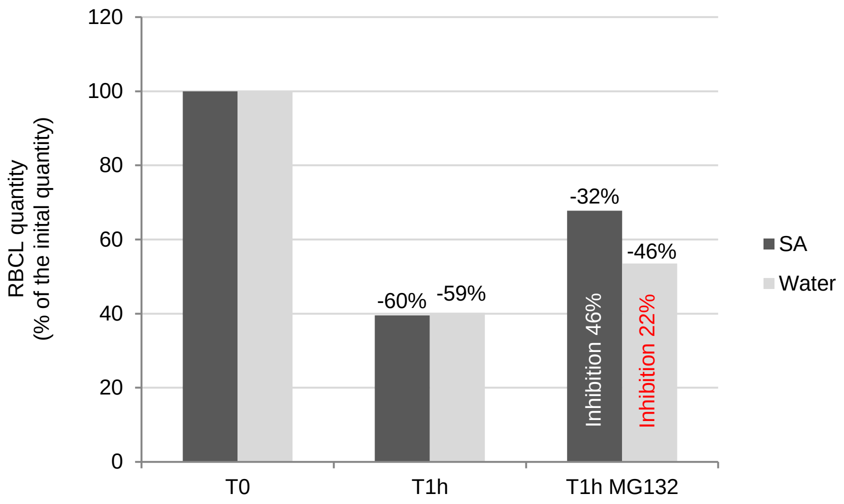

Figure S2 I *In vitro* degradation of the large subunit of RuBisCO in absence or presence of MG132 (inhibitor of cysteine proteases and the proteasome) after infiltration of salicylic acid or water into mature leaves of *Brassica napus* L. (cv. Aviso)

Mature leaves of oilseed rape were infiltrated with water or 1mM of salicylic acid (SA). Soluble proteins were extracted 96h after infiltrations. Samples were incubated in sodium acetate buffer (50 mM, pH 5.5) for 1h at 37°C without inhibitor or in presence of 0,95 mg/mL of MG132 (inhibitor of cysteine proteases and the proteasome activities). The reaction was stopped by adding ice-cold acetone. To determine the initial quantity of RuBisCo (large subunit, RBCL), another sample was also treated as described above but the reaction was stopped immediately. Samples were separated on SDS-PAGE Stain-free gels (Mini-PROTEAN® TGX™ Stain Free), scanned under UV light with a Gel Doc™ EZ scanner and analysed. The degradation of RBCL was calculated as the difference in quantity between non incubated and incubated samples and expressed as % of RBCL degradation while the % of inhibition due to the MG132 was calculated as the difference in degradation in the presence or absence of the inhibitor. Aviso was used for this experiment because, like Tenor, this genotype is characterized by an increase in N use efficiency and leaf N remobilization compared to high N that is sufficient to sustain the leaf biomass production at a similar level than non-limited plants (Girondé et al., 2015).

Table S3 I Identification by LC-MS/MS of serine proteases labelled with the biotin-tagged probe FP in senescent leaves of *Brassica napus* L. (cv. Ténor) after 23 days of LN treatment. To characterize SPs observed in **Figure 3**, labelling with FP-biotin carrying a biotin tag was performed followed by a pull-down of biotinylated proteins (**Fig. S1**, for details see Material and Methods). The detected zones were excised, treated and analysed by LC-MS/MS. LC-MS/MS spectra were searched against the *Viridiplantae* database focused on *Brassica napus* L. using MASCOT 2.5.0 and only scores higher or equal to 48 were considered. The assigned protein of best match is provided alongside the UniProt or NCBI/GenBank accession number. Scores, queries matched, peptide matches, different peptide matches, experimental mass and theoretical mass are also presented. Other proteins identified in *Brassica napus* with the same peptides by MASCOT are also presented. Protein sequences were matched against sequences of Brassicacea proteins using the NCBI Blast Protein Database (algorithm blastp) and better results of blasts are presented with the name of the protein, the organism, the UniProt or NCBI/GenBank accession number and the percentage of sequence identity. Finally, SPs were classified according to the MEROPS database.

Cutting zone (kDa)	Protein accession no [*Brassica napus*] / Uniprot or NCBI accession no.	Score	Queries Matched	Peptides matched	Different matched peptides	Exp. Mass (Da)	Theo. Mass (Da)	Theo. pI	Others proteins identified with the same peptides by MASCOT	Result of BLAST Protein [*Brassica napus* or other organism]- Protein [Brassica napus] / Uniprot or NCBI accession no. / % identity	Classification
1 (70)	Acylamino-acid-releasing enzyme-like isoform X1 [*Brassica napus*] / XP_013655680.1	630	35	24	23	89982	90038,97	6,16	Acylamino-acid-releasing enzyme-like isoform X2 [*Brassica napus*] / XP_013655681.1	BnaA08g30180D [*Brassica napus*] / A0A078IWH3 / 97 %	POPLs S9
	BnaA08g30180D [*Brassica napus*] / A0A078IWH3	545	30	21	20	89758	89814,83	6,24	-	Acylamino-acid-releasing enzyme-like isoform X1 [*Brassica napus*] / XP_013655680.1 / 97 %	POPLs S9
	BnaCnng64630D [*Brassica napus*] / A0A078JVT3	406	23	19	18	81782	81844,29	5,35	Prolyl endopeptidase-like [*Brassica napus*] / XP_013693488.1	Prolyl endopeptidase-like [*Brassica napus*] / XP_013693488.1 / 99 %	POPLs S9
	BnaCnng28400D [*Brassica napus*] / A0A078J055	377	21	19	18	81798	81850,38	5,39	-	Prolyl endopeptidase-like [*Brassica napus*] / XP_013676746.1 / 98 %	POPLs S9
	Prolyl endopeptidase-like [*Brassica napus*] / XP_013656097.1	241	20	17	16	90275	90331,87	5,65	-	Prolyl endopeptidase-like [*Brassica napus*] / XP_013717916.1 / 99 %	POPLs S9
	BnaC06g11680D [*Brassica napus*] / CDY06476.1	241	12	11	11	90471	90528,61	5,56	Acylamino-acid-releasing enzyme-like isoform X2 [*Brassica napus*] / XP_013697439.1	Acylamino-acid-releasing enzyme-like isoform X2 [*Brassica napus*] / XP_013702333.1 / 99 %	POPLs S9
	BnaA09g50980D [*Brassica napus*] / A0A078H6S6	229	10	10	10	125156	125234,8	5,98	Methylcrotonoyl-CoA carboxylase subunit alpha, mitochondrial isoform X1 [*Brassica napus*] / XP_013664865.1	Methylcrotonoyl-CoA carboxylase subunit alpha, mitochondrial isoform X1 [*Brassica napus*] / XP_013664865.1 / 99 %	-
	BnaC08g45780D [*Brassica napus*] / A0A078HGH7	216	10	10	10	124850	124928,4	6,1	Methylcrotonoyl-CoA carboxylase subunit alpha, mitochondrial-like isoform X1 [*Brassica napus*] / XP_013701757.1	Methylcrotonoyl-CoA carboxylase subunit alpha, mitochondrial-like isoform X1 [*Brassica napus*] / XP_013701757.1 / 100 %	-
	Subtilisin-like protease SBT1.7 [*Brassica napus*] / XP_013654072.1	162	9	8	8	73353	73398,89	7,57	Subtilisin-like protease SBT1.7 [*Brassica napus*] / XP_013655824.1	Subtilisin-like protease SBT1.7 [*Brassica napus*] / XP_013655824.1 / 100 %	Subtilisins S8
	BnaCnng41800D [*Brassica napus*] / A0A078JDZ2	139	6	6	6	76219	76267,11	6,5	Subtilisin-like protease SBT1.7 [*Brassica napus*] / XP_013655823.1	Subtilisin-like protease SBT1.7 [*Brassica napus*] / XP_013655823.1 / 95 %	Subtilisins S8
	Acylamino-acid-releasing enzyme-like [*Brassica napus*] / XP_013644416.1	103	6	4	4	82915	82967,26	6,39	-	BnaA01g19230D [*Brassica napus*] / A0A078GYS9 / 98 % PREDICTED: acylamino-acid-releasing enzyme [*Brassica oleracea var. oleracea*] / XP_013624955.1 / 99 %	POPLs S9
	Prolyl endopeptidase-like [*Brassica napus*] / XP_013642253.1	83	7	6	6	82713	82765,56	5,16	-	BnaA06g14380D [*Brassica napus*] / A0A078GWK8 / 98 % PREDICTED: prolyl endopeptidase-like [*Brassica oleracea var. oleracea*] / XP_013587623.1 / 99 %	POPLs S9

Table S3 I (Continued)

Cutting zone (kDa)	Protein accession no [*Brassica napus*] / Uniprot or NCBI accession no.	Score	Queries Matched	Peptides matched	Different matched peptides	Exp. Mass (Da)	Theo. Mass (Da)	Theo. pI	Others proteins identified with the same peptides by MASCOT	Result of BLAST Protein [*Brassica napus* or other organism] / Uniprot or NCBI accession no. / % identity	Classification
2 (50)	BnaA06g18620D [*Brassica napus*] / CDX99171.1	338	28	28	17	56843	56878,91	5,35	-	Serine carboxypeptidase-like 47 [*Brassica napus*] / XP_013680609.1 / 69 %	S10
3 (45)	BnaA06g19010D [*Brassica napus*] / A0A078HJQ8	82	4	4	4	35528	35550,37	5,16	Probable carboxylesterase 12 [*Brassica napus*] / XP_013701961.1	Probable carboxylesterase 12 [*Brassica napus*] /XP_013701961.1 / 98 %	CXEs
	BnaC04g00530D [*Brassica napus*] / A0A078HW24	74	3	3	3	45594	45623,8	7,79	Pectin acetylesterase 3-like [*Brassica napus*]/ XP_013747575.1 ; Pectin acetylesterase 3-like [*Brassica napus*] / XP_013686140.1	Pectin acetylesterase 3-like [*Brassica napus*] / XP_013747575.1 / 98 %	PAEs
	BnaC01g38630D [*Brassica napus*] / A0A078J0P9	69	5	5	3	67210	67253,35	5,31	Serine carboxypeptidase-like 49 [*Brassica napus*] / XP_013676539.1	Serine carboxypeptidase-like 49 [*Brassica napus*] / XP_013676539.1 / 97 %	S10
4 (37)	BnaA06g19010D [*Brassica napus*] / A0A078HJQ8	668	95	25	16	35528	35550,37	5,16	Probable carboxylesterase 12 [*Brassica napus*] / XP_013701961.1	Probable carboxylesterase 12 [*Brassica napus*] /XP_013701961.1 / 98 %	CXEs
	PREDICTED: probable carboxylesterase 12, partial [*Brassica napus*] / XP_013704469.1	500	52	16	14	29451	29469,5	5,38	-	PREDICTED: probable carboxylesterase 12 [*Brassica napus*] / XP_013701961.1 / 98 %	CXEs
	BnaC03g52990D [*Brassica napus*] / CDX77920.1	467	55	16	14	34316	34337,89	5,23	-	Probable carboxylesterase 12 [*Brassica napus*] / XP_013701961.1 / 95 %	CXEs
	Protease Do-like 1, chloroplastic [*Brassica napus*] / XP_013644609.1	263	12	10	10	46206	46234,66	6	-	PREDICTED: protease Do-like 1, chloroplastic [*Brassica rapa*] / XP_009151909.1 / 99 %	Deg S1
	Serine carboxypeptidase-like 35 [*Brassica napus*] / XP_013701943.1	82	4	4	4	54114	54148,29	6,83	-	Serine carboxypeptidase-like 35 [*Brassica napus*] / XP_013715466.1 / 96 %	S10
	BnaA01g20450D [*Brassica napus*] / CDX83045.1	71	12	2	2	35650	35672,84	5,32	Probable carboxylesterase 12 [*Brassica napus*] / XP_013745017.1 ; Probable carboxylesterase 12 [*Brassica napus*] / XP_013670118.1	PREDICTED: probable carboxylesterase 12 [*Brassica napus*] / XP_013745017.1 / 100 %	CXEs

Table S3 I (Continued)

Cutting zone (kDa)	Protein accession no [*Brassica napus*] / Uniprot or NCBI accession no.	Score	Queries Matched	Peptides matched	Different matched peptides	Exp. Mass (Da)	Theo. Mass (Da)	Theo. pI	Others proteins identified with the same peptides by MASCOT	Result of BLAST Protein [*Brassica napus* or other organism] / Uniprot or NCBI accession no. / % identity	Classification
5 (35)	BnaA04g07190D [*Brassica napus*] / CDY39716.1	184	13	6	5	56619	56655,77	6,21	BnaC04g29380D [*Brassica napus*] / A0A078HA31 ; PREDICTED: serine carboxypeptidase-like 20 [*Brassica napus*] / XP_013696030.1	BnaC04g29380D [*Brassica napus*] / A0A078HA31 / 100 % ; PREDICTED: serine carboxypeptidase-like 20 [*Brassica napus*] /XP_013696030.1/ 91 %	S10
	BnaC04g01950D [*Brassica napus*] / A0A078GFD5	171	8	4	4	31524	31544,48	5,84	-	S-formylglutathione hydrolase-like [*Brassica napus*] / XP_013747828.1 / 98 %	-
	BnaA03g19510D [*Brassica napus*] / A0A078HD96	165	10	4	4	31869	31888,93	6,24	-	S-formylglutathione hydrolase [*Brassica napus*] / XP_013734472.1 / 99 %	-
	BnaA06g19010D [*Brassica napus*] / A0A078HJQ8	150	9	8	8	35528	35550,37	5,16	Probable carboxylesterase 12 [*Brassica napus*] / XP_013701961.1	Probable carboxylesterase 12 [*Brassica napus*] /XP_013701961.1 / 98 %	CXEs
	PREDICTED: probable carboxylesterase 12, partial [*Brassica napus*] / XP_013704469.1	97	8	7	7	29451	29469,5	5,38	-	PREDICTED: probable carboxylesterase 12 [*Brassica napus*] / XP_013701961.1 / 98 %	CXEs
	BnaA01g06330D [*Brassica napus*] / A0A078GRW	95	3	2	2	53488	53521,41	5,43	Serine carboxypeptidase-like 29 [*Brassica napus*] / XP_013670649.1	Serine carboxypeptidase-like 29 [*Brassica napus*] / XP_013670751.1 / 99 %	S10
	BnaA02g33270D [*Brassica napus*] / A0A078GW60	87	5	5	5	17414	17424,86	6,5	-	PREDICTED: probable carboxylesterase 120 [*Brassica rapa*] / XP_009130199.1 / 100 %	CXEs
	S-formylglutathione hydrolase-like [*Brassica napus*] / XP_013747828.1	74	5	3	3	31438	31458,38	5,57	-	BnaC04g01950D [*Brassica napus*] / A0A078GFD5 / 98 % PREDICTED: S-formylglutathione hydrolase-like [*Brassica rapa*] / XP_009142750.1 / 98 %	-
	Serine carboxypeptidase-like 35 [*Brassica napus*] / XP_013701943.1	68	5	4	4	54114	54148,29	6,83	-	Serine carboxypeptidase-like 35 [*Brassica napus*] / XP_013715466.1 / 96 %	S10

Table S3 I (Continued)

Cutting zone (kDa)	Protein accession no [*Brassica napus*] / Uniprot or NCBI accession no.	Score	Queries Matched	Peptides matched	Different matched peptides	Exp. Mass (Da)	Theo. Mass (Da)	Theo. pI	Others proteins identified with the same peptides by MASCOT	Result of BLAST Protein [*Brassica napus* or other organism] / Uniprot or NCBI accession no. / % identity	Classification
6 (30)	BnaA04g07190D [*Brassica napus*] / CDY39716.1	161	17	17	8	56619	56655,77	6,21	-	BnaC04g29380D [*Brassica napus*] / A0A078HA31 / 100 % ; PREDICTED: serine carboxypeptidase-like 20 [*Brassica napus*] /XP_013696030.1/ 91 %	S10
	Serine carboxypeptidase-like 35 [*Brassica napus*] / XP_013701943.1	129	9	9	6	54114	54148,29	6,83	-	Serine carboxypeptidase-like 35 [*Brassica napus*] / XP_013715466.1 / 96 %	S10
	BnaA06g19010D [*Brassica napus*] / A0A078HJQ8	123	7	7	5	35528	35550,37	5,16	Probable carboxylesterase 12 [*Brassica napus*] / XP_013701961.1	Probable carboxylesterase 12 [*Brassica napus*] /XP_013701961.1 / 98 %	CXEs
	BnaA01g06330D [*Brassica napus*] / A0A078GRW	114	11	11	5	53488	53521,41	5,43	Serine carboxypeptidase-like 29 [*Brassica napus*] / XP_013670649.1	Serine carboxypeptidase-like 29 [*Brassica napus*] / XP_013670751.1 / 99 %	S10
	Serine carboxypeptidase-like 29 [*Brassica napus*] / XP_013750696.1	110	4	4	3	53001	53035	6,02	-	Serine carboxypeptidase-like 29 [*Brassica napus*] / XP_013720411.1 / 98 %	S10
	BnaA08g12880D [*Brassica napus*] / A0A078GF58	100	4	4	3	53131	53164,05	5,87	-	Serine carboxypeptidase-like 29 [*Brassica napus*] / XP_013720411.1 / 99 %	S10
	BnaA03g19510D [*Brassica napus*] / A0A078HD96	81	4	4	4	31869	31888,93	6,24	-	S-formylglutathione hydrolase [*Brassica napus*] / XP_013734472.1 / 99 %	-
	PREDICTED: probable carboxylesterase 12, partial [*Brassica napus*] / XP_013704469.1	77	5	5	4	29451	29469,5	5,38	-	PREDICTED: probable carboxylesterase 12 [*Brassica napus*] / XP_013701961.1 / 98 %	CXEs
	Lysosomal Pro-X carboxypeptidase-like [*Brassica napus*] / XP_013643207.1	68	2	2	2	57149	57185,35	4,94	Lysosomal Pro-X carboxypeptidase-like [*Brassica napus*] / XP_013643319.1	Lysosomal Pro-X carboxypeptidase-like [*Brassica napus*] / XP_013643319.1 / 97 %	S10
	BnaC04g01950D [*Brassica napus*] / A0A078GFD5	63	4	4	4	31524	31544,48	5,84	-	S-formylglutathione hydrolase-like [*Brassica napus*] / XP_013747828.1 / 98 %	-
7 (27)	BnaA04g07190D [*Brassica napus*] / CDY39716.1	162	10	10	6	56619	56655,77	6,21	BnaC04g29380D [*Brassica napus*] / A0A078HA31 ; PREDICTED: serine carboxypeptidase-like 20 [*Brassica napus*] / XP_013696030.1	BnaC04g29380D [*Brassica napus*] / A0A078HA31 / 100 % ; PREDICTED: serine carboxypeptidase-like 20 [*Brassica napus*] /XP_013696030.1/ 91 %	S10
	BnaA01g06330D [*Brassica napus*] / A0A078GRW	141	13	13	5	53488	53521,41	5,43	Serine carboxypeptidase-like 29 [*Brassica napus*] / XP_013670649.1	Serine carboxypeptidase-like 29 [*Brassica napus*] / XP_013670751.1 / 99 %	S10
	BnaA04g16130D [*Brassica napus*] / A0A078GVN3	133	4	4	4	51093	51125,51	6,74	Serine carboxypeptidase-like 51 [*Brassica napus*] / XP_013743962.1 ; Serine carboxypeptidase-like 51 [*Brassica napus*] / XP_013745961.1	Serine carboxypeptidase-like 51 [*Brassica napus*] / XP_013743970.1 / 99 %	S10
	BnaA06g19010D [*Brassica napus*] / A0A078HJQ8	82	4	4	4	35528	35550,37	5,16	Probable carboxylesterase 12 [*Brassica napus*] / XP_013701961.1	Probable carboxylesterase 12 [*Brassica napus*] /XP_013701961.1 / 98 %	CXEs
	PREDICTED: probable carboxylesterase 12, partial [*Brassica napus*] / XP_013704469.1	67	4	4	4	29451	29469,5	5,38	-	PREDICTED: probable carboxylesterase 12 [*Brassica napus*] / XP_013701961.1 / 98 %	CXEs

Table S3 I (Continued)

Cutting zone (kDa)	Protein accession no [*Brassica napus*] / Uniprot or NCBI accession no.	Score	Queries Matched	Peptides matched	Different matched peptides	Exp. Mass (Da)	Theo. Mass (Da)	Theo. pI	Others proteins identified with the same peptides by MASCOT	Result of BLAST Protein [*Brassica napus* or other organism] / Uniprot or NCBI accession no. / % identity	Classification
8 (25)	BnaA01g06330D [*Brassica napus*] / A0A078GRW	350	23	10	8	53488	53521,41	5,43	Serine carboxypeptidase-like 29 [*Brassica napus*] / XP_013670649.1	Serine carboxypeptidase-like 29 [*Brassica napus*] / XP_013670751.1 / 99 %	S10
	BnaA06g19010D [*Brassica napus*] / A0A078HJQ8	340	20	13	13	35528	35550,37	5,16	Probable carboxylesterase 12 [*Brassica napus*] / XP_013701961.1	Probable carboxylesterase 12 [*Brassica napus*] /XP_013701961.1 / 98 %	CXEs
	Methylesterase 9-like isoform X1 [*Brassica napus*] / XP_013742536.1	238	12	9	9	28933	28952,42	5,11	-	Methylesterase 9-like isoform X2 [*Brassica napus*] / XP_013746536.1 / 92 %	MESs
	PREDICTED: probable carboxylesterase 12, partial [*Brassica napus*] / XP_013704469.1	236	14	9	9	29451	29469,5	5,38	-	PREDICTED: probable carboxylesterase 12 [*Brassica napus*] / XP_013701961.1 / 98 %	CXEs
	BnaAnng04870D [*Brassica napus*] / A0A078HJH5	159	6	6	6	29281	29299,67	7,66	-	BnaC02g43760D [*Brassica napus*] / A0A078H800 / 96 % PREDICTED: ovarian cancer-associated gene 2 protein homolog [*Brassica rapa*] / XP_009130510.1 / 99 %	-
	BnaA10g23100D [*Brassica napus*] / CDX69961.1	141	9	5	5	53907	53941,01	6,43	-	Serine carboxypeptidase-like 35 [*Brassica napus*] / XP_013715466.1 / 99 %	S10
	Methylesterase 9-like isoform X1 [*Brassica napus*] / XP_013746535.1	139	7	6	6	29068	29087,55	4,89	Methylesterase 9-like isoform X2 [*Brassica napus*] / XP_013746536.1	Methylesterase 9-like isoform X2 [*Brassica napus*] / XP_013746536.1/ 99 %	MESs
	BnaC06g11680D [*Brassica napus*] / CDY06476.1	118	4	4	4	90471	90528,61	5,56	Acylamino-acid-releasing enzyme-like isoform X2 [*Brassica napus*] / XP_013697439.1	Acylamino-acid-releasing enzyme-like isoform X2 [*Brassica napus*] / XP_013702333.1 / 99 %	POPLs S9
	BnaC07g37240D [*Brassica napus*] / CDX94090.1		6	4	4	28565	28583,76	5,58	-	BnaA03g45270D [*Brassica napus*] / CDX98742.1 / 94 % PREDICTED: probable carboxylesterase SOBER1-like isoform X1 [*Brassica oleracea var. oleracea*] / XP_013593386.1 / 98 %	CXEs
	BnaC04g36040D [*Brassica napus*] / CDX89585.1	113	6	5	5	27059	27077,27	5,69	-	BnaA04g13790D [*Brassica napus*] /A0A078HQA8 / 89 % PREDICTED: methylesterase 1 [*Brassica oleracea var. oleracea*] / XP_013634667.1 / 92 %	MESs
	Acyl-protein thioesterase 2-like [*Brassica napus*] / XP_013730870.1	105	5	4	4	26707	26723,43	6,15	Acyl-protein thioesterase 2-like [*Brassica napus*] /XP_013730875.1	Acyl-protein thioesterase 2-like [*Brassica napus*] / XP_013730875.1 / 99 %	-
	BnaA08g12880D [*Brassica napus*] / A0A078GF58	104	5	4	4	53131	53164,05	5,87	-	Serine carboxypeptidase-like 29 [*Brassica napus*] / XP_013720411.1 / 99 %	S10
	Serine carboxypeptidase-like 35 [*Brassica napus*] / XP_013701943.1	103	7	4	4	54114	54148,29	6,83	-	Serine carboxypeptidase-like 35 [*Brassica napus*] / XP_013715466.1 / 96 %	S10
	BnaA01g00900D [*Brassica napus*] / CDX75612.1	102	4	4	4	28614	28632,94	5,23	Methylesterase 9-like [*Brassica napus*] / XP_013734088.1	Methylesterase 9-like [*Brassica napus*] / XP_013734088.1 / 99 %	MESs
	Lysosomal Pro-X carboxypeptidase-like [*Brassica napus*] / XP_013643207.1	100	5	3	3	57149	57185,35	4,94	-	Lysosomal Pro-X carboxypeptidase-like [*Brassica napus*] / XP_013643319.1 / 97 %	S10
										BnaA01g00900D [*Brassica napus*] / CDX75612.1 / 96 %	

Table S4 I Identification by LC-MS/MS of PLCPs labelled with the biotin-tagged probe DCG04 in a senescing leaf of *Brassica napus* L. (cv. Ténor) after 23 days of LN treatment. To characterize PLCPs observed in **Figure 4**, labelling with DCG04 carrying a biotin tag was performed followed by a pull-down of biotinylated proteins (**Fig. S1**, for details see Material and Methods). The detected zones were excised, treated and analysed by LC-MS/MS. LC-MS/MS spectra were searched against the *Viridiplantae* database focused on *Brassica napus* L. using MASCOT 2.5.0 and only scores higher or equal to 48 were considered. The assigned protein of best match is provided alongside the UniProt or NCBI/GenBank accession number. Scores, queries matched, peptide matches, different peptide matches, experimental mass and theoretical mass are also presented. Other proteins identified in *Brassica napus* with the same peptides by MASCOT are also presented. Protein sequences were matched against sequences of Brassicacea proteins using the NCBI BLAST Protein Database (algorithm blastp) and better results of BLASTS are presented with the name of the protein, the organism, the UniProt or NCBI/GenBank accession number and the percentage of sequence identity. Finally, PLCPs were classified according to the classification of Richau et al. (2012).

Cutting zone (kDa)	Protein accession no [*Brassica napus*] / Uniprot or NCBI accession no.	Score	Queries Matched	Peptides matched	Different matched peptides	Exp. Mass (Da)	Theo. Mass (Da)	Theo. pI	Others proteins identified with the same peptides by MASCOT	Result of BLAST Protein [*Brassica napus* or other organism] / Uniprot or NCBI accession no. / % identity	Classification
1 (40)	BnaA10g05390D [*Brassica napus*] / CDY06760.1	425	41	41	13	50452	50484,76	5,3	-	Cysteine proteinase RD21a [*Brassica napus*] / XP_013718810.1 / 97 %	RD21-like
	BnaA08g04080D [*Brassica napus*] / A0A078FVG4	336	31	31	12	51069	50657,95	5,23	PREDICTED: cysteine proteinase RD21a-like [*Brassica napus*] /XP_013667423.1; Cysteine proteinase RD21a-like [*Brassica napus*] / XP_013701858.1	PREDICTED: cysteine proteinase RD21a-like [*Brassica napus*] / XP_013667423.1 / 99 %	RD21-like
	PREDICTED: cysteine proteinase RD21a [*Brassica napus*] / XP_013718810.1	301	37	37	12	50534	50566,84	5,23	-	BnaA10g05390D [*Brassica napus*] /CDY06760.1 / 97 % senescence-associated cysteine protease [*Brassica oleracea*] / Q8W180 / 94 %	RD21-like
	BnaA06g36920D [*Brassica napus*] /A0A078G7A3	136	6	6	5	50506	50539,07	5,57	Probable cysteine protease RD21B [*Brassica napus*] / XP_013644992 ; Probable cysteine protease RD21B [*Brassica napus*] / XP_013646239.1 ; Probable cysteine protease RD21B isoform X1 [*Brassica napus*] / XP_013692214.1 ; probable cysteine protease RD21B isoform X2 [*Brassica napus*] / XP_013692215.1	Probable cysteine protease RD21B [*Brassica napus*] / XP_013644992.1 / 99 %	RD21-like
	senescence-specific cysteine protease [*Brassica napus*] / Q9SQH3	48	3	13	1	38333	38357,54	6,99	-	Senescence-specific cysteine protease SAG12-like [*Brassica napus*] / XP_013677175.1 / 99 %	SAG12-like

Table S4 I (Continued)

Cutting zone (kDa)	Protein accession no [*Brassica napus*] / Uniprot or NCBI accession no.	Score	Queries Matched	Peptides matched	Different matched peptides	Exp. Mass (Da)	Theo. Mass (Da)	Theo. pI	Others proteins identified with the same peptides by MASCOT	Result of BLAST Protein [*Brassica napus* or other organism] / Uniprot or NCBI accession no. / % identity	Classification
2 (35)	BnaA10g05390D [*Brassica napus*] / CDY06760.1	308	11	11	8	50452	50484,76	5,3	-	Cysteine proteinase RD21a [*Brassica napus*] / XP_013718810.1 / 97 %	RD21-like
	BnaA06g36920D [*Brassica napus*] /A0A078G7A3	269	11	11	7	50506	50539,07	5,57	Probable cysteine protease RD21B [*Brassica napus*] / XP_013644992 ; Probable cysteine protease RD21B [*Brassica napus*] / XP_013646239.1 ; Probable cysteine protease RD21B isoform X1 [*Brassica napus*] / XP_013692214.1 ; probable cysteine protease RD21B isoform X2 [*Brassica napus*] / XP_013692215.1	Probable cysteine protease RD21B [*Brassica napus*] / XP_013644992.1 / 99 %	RD21-like
	BnaA08g04080D [*Brassica napus*] / A0A078FVG4	209	11	11	6	50625	50657,95	5,23	PREDICTED: cysteine proteinase RD21a-like [*Brassica napus*] /XP_013667423.1; Cysteine proteinase RD21a-like [*Brassica napus*] / XP_013701858.1	PREDICTED: cysteine proteinase RD21a-like [*Brassica napus*] / XP_013667423.1 / 99 %	RD21-like
	PREDICTED: cysteine proteinase RD21a [*Brassica napus*] / XP_013718810.1	186	8	8	6	50534	50566,84	5,23	-	BnaA10g05390D [*Brassica napus*] /CDY06760.1 / 97 % senescence-associated cysteine protease [*Brassica oleracea*] / Q8W180 / 94 %	RD21-like
	senescence-specific cysteine protease [*Brassica napus*] / Q9SQH3	75	4	4	4	38333	38357,54	6,99	-	Senescence-specific cysteine protease SAG12-like [*Brassica napus*] / XP_013677175.1 / 99 %	SAG12-like

Table S4 I (Continued)

Cutting zone (kDa)	Protein accession no [*Brassica napus*] / Uniprot or NCBI accession no.	Score	Queries Matched	Peptides matched	Different matched peptides	Exp. Mass (Da)	Theo. Mass (Da)	Theo. pI	Others proteins identified with the same peptides by MASCOT	Result of BLAST Protein [*Brassica napus* or other organism] / Uniprot or NCBI accession no. / % identity	Classification
3 (30)	BnaA10g05390D [*Brassica napus*] / CDY06760.1	239	11	11	7	50452	50484,76	5,3	-	Cysteine proteinase RD21a [*Brassica napus*] / XP_013718810.1 / 97 %	RD21-like
	BnaA08g04080D [*Brassica napus*] / A0A078FVG4	181	11	11	6	51069	50657,95	5,23	PREDICTED: cysteine proteinase RD21a-like [*Brassica napus*] /XP_013667423.1; Cysteine proteinase RD21a-like [*Brassica napus*] / XP_013701858.1	PREDICTED: cysteine proteinase RD21a-like [*Brassica napus*] / XP_013667423.1 / 99 %	RD21-like
	PREDICTED: cysteine proteinase RD21a [*Brassica napus*] / XP_013718810.1	131	7	7	5	50534	50566,84	5,23	-	BnaA10g05390D [*Brassica napus*] /CDY06760.1 / 97 % senescence-associated cysteine protease [*Brassica oleracea*] / Q8W180 / 94 %	RD21-like
	BnaA06g36920D [*Brassica napus*] /A0A078G7A3	109	4	4	3	50506	50539,07	5,57	Probable cysteine protease RD21B [*Brassica napus*] / XP_013644992 ; Probable cysteine protease RD21B [*Brassica napus*] / XP_013646239.1 ; Probable cysteine protease RD21B isoform X1 [*Brassica napus*] / XP_013692214.1 ; probable cysteine protease RD21B isoform X2 [*Brassica napus*] / XP_013692215.1	Probable cysteine protease RD21B [*Brassica napus*] / XP_013644992.1 / 99 %	RD21-like
	BnaCnng01440D [*Brassica napus*] / CDY07129.1	95	3	3	3	39080	39105,37	5,92	Cathepsin B-like [*Brassica napus*] / XP_013722833.1	Cathepsin B-like protease 3 [*Brassica napus*] / XP_013722833.1 / 99 %	Cathepsin B-like
	Cysteine proteinase RD19a-like [*Brassica napus*] / XP_013701923.1	79	3	3	3	40117	40142,41	7,52	-	Cysteine proteinase RD19a-like [*Brassica napus*] / XP_013656686.1 / 95 %	RD19-like
	Cysteine proteinase RD19a [*Brassica napus*] / XP_013747708.1	78	3	3	3	39960	39985,15	6,32	Cysteine proteinase RD19a-like [*Brassica napus*] /XP_013735608.1 ; BnaA01g05320D [*Brassica napus*] / A0A078HTU1	BnaA01g05320D [*Brassica napus*] / A0A078HTU1 / 99 % Cysteine proteinase RD19a-like [*Brassica napus*] / > XP_013735608.1 / 98 %	RD19-like
	BnaA09g52180D [*Brassica napus*] / A0A078J5J7	77	3	3	3	38949	38974,14	5,78	-	BnaCnng01440D [*Brassica napus*] / CDY07129.1 / 97 % PREDICTED: cathepsin B [*Brassica rapa*] / XP_009111352.1 / 99 %	Cathepsin B
	senescence-specific cysteine protease [*Brassica napus*] / Q9SQH3	65	5	5	4	38333	38357,54	6,99	-	Senescence-specific cysteine protease SAG12-like [*Brassica napus*] / XP_013677175.1 / 99 %	SAG12-like

Table S4 I (Continued)

Cutting zone (kDa)	Protein accession no [*Brassica napus*] / Uniprot or NCBI accession no.	Score	Queries Matched	Peptides matched	Different matched peptides	Exp. Mass (Da)	Theo. Mass (Da)	Theo. pI	Others proteins identified with the same peptides by MASCOT	Result of BLAST Protein [*Brassica napus* or other organism] / Uniprot or NCBI accession no. / % identity	Classification
4 (27)	BnaA08g04080D [*Brassica napus*] / A0A078FVG4	155	5	5	5	50625	50657,95	5,23	PREDICTED: cysteine proteinase RD21a-like [*Brassica napus*] /XP_013667423.1; Cysteine proteinase RD21a-like [*Brassica napus*] / XP_013701858.1	PREDICTED: cysteine proteinase RD21a-like [*Brassica napus*] / XP_013667423.1 / 99 %	RD21-like
	BnaA10g05390D [*Brassica napus*] / CDY06760.1	126	6	5	5	50452	50484,76	5,3	-	Cysteine proteinase RD21a [*Brassica napus*] / XP_013718810.1 / 97 %	RD21-like
	BnaA09g52180D [*Brassica napus*] / A0A078J5J7	120	3	3	3	38949	38974,14	5,78	-	BnaCnng01440D [*Brassica napus*] / CDY07129.1 / 97 % PREDICTED: cathepsin B [*Brassica rapa*] / XP_009111352.1 / 99 %	Cathepsin B
	PREDICTED: cysteine proteinase RD21a [*Brassica napus*] / XP_013718810.1	110	3	5	5	50534	50566,84	5,23	-	BnaA10g05390D [*Brassica napus*] /CDY06760.1 / 97 % senescence-associated cysteine protease [*Brassica oleracea*] / Q8W180 / 94 %	RD21-like
	senescence-specific cysteine protease [*Brassica napus*] / Q9SQH3	100	8	7	5	38333	38357,54	6,99	-	Senescence-specific cysteine protease SAG12-like [*Brassica napus*] / XP_013677175.1 / 99 %	SAG12-like
	BnaCnng01440D [*Brassica napus*] / CDY07129.1	98	2	2	2	39080	39105,37	5,92	Cathepsin B-like [*Brassica napus*] / XP_013722833.1	Cathepsin B-like protease 3 [*Brassica napus*] / XP_013722833.1 / 99 %	Cathepsin B-like
	Cysteine proteinase RD19a-like [*Brassica napus*] / XP_013701923.1	51	2	2	2	40117	40142,41	7,52	-	Cysteine proteinase RD19a-like [*Brassica napus*] /XP_013656686.1 / 95 %	RD19-like
	BnaA06g40240D [*Brassica napus*] / A0A078J304	48	4	3	3	35458	35480,31	9,01	Senescence-specific cysteine protease SAG12-like [*Brassica napus*] / XP_013697317.1 ; senescence-specific cysteine protease SAG12-like precursor [*Brassica napus*] / NP_001302502.1	Senescence-specific cysteine protease SAG12-like [*Brassica napus*] / XP_013697317.1 / 93 %	SAG12-like

Table S4 I (Continued)

Cutting zone (kDa)	Protein accession no [*Brassica napus*] / Uniprot or NCBI accession no.	Score	Queries Matched	Peptides matched	Different matched peptides	Exp. Mass (Da)	Theo. Mass (Da)	Theo. pI	Others proteins identified with the same peptides by MASCOT	Result of BLAST Protein [*Brassica napus* or other organism] / Uniprot or NCBI accession no. / % identity	Classification
5 (25)	Low-temperature-induced cysteine proteinase-like isoform X2 [*Brassica napus*] /XP_013696759.1	295	18	11	9	48551	48582,78	6,27	-	BnaA06g05780D [*Brassica napus*] / CDX93470.1 / 97 % PREDICTED: low-temperature-induced cysteine proteinase [*Brassica oleracea var. oleracea*] / XP_013587201.1 / 99 %	XBCP3-like
	BnaA06g05780D [*Brassica napus*] / CDX93470.1	260	18	11	10	48789	48821,05	6,59	-	Low-temperature-induced cysteine proteinase-like isoform X2 [*Brassica napus*] / XP_013696759.1 / 97 %	XBCP3-like
	BnaC09g35690D [*Brassica napus*] / CDX80173.1	198	16	9	7	39265	39290,37	5,57	-	thiol protease aleurain precursor [*Brassica napus*] / NP_001302920.1 / 97 %	AALP-like
	BnaA10g05390D [*Brassica napus*] / CDY06760.1	160	8	5	5	50452	50484,76	5,3	-	Cysteine proteinase RD21a [*Brassica napus*] / XP_013718810.1 / 97 %	RD21-like
	PREDICTED: cysteine proteinase RD21a [*Brassica napus*] / XP_013718810.1	159	7	5	5	50534	50566,84	5,23	-	BnaA10g05390D [*Brassica napus*] /CDY06760.1 / 97 % senescence-associated cysteine protease [*Brassica oleracea*] / Q8W180 / 94 %	RD21-like
	BnaC01g26060D [*Brassica napus*] / CDX68528.1	135	14	5	5	38107	38131,6	5,21	Zingipain-2-like [*Brassica napus*] / XP_013749848.1	Zingipain-2-like [*Brassica napus*] / XP_013749848.1 / 98 %	-
	BnaA08g04080D [*Brassica napus*] / A0A078FVG4	115	6	4	4	50625	50657,95	5,23	PREDICTED: cysteine proteinase RD21a-like [*Brassica napus*] /XP_013667423.1; Cysteine proteinase RD21a-like [*Brassica napus*] / XP_013701858.1	PREDICTED: cysteine proteinase RD21a-like [*Brassica napus*] / XP_013667423.1 / 99 %	RD21-like
	BnaA06g40240D [*Brassica napus*] / A0A078J304	75	16	3	3	35458	35480,31	9,01	Senescence-specific cysteine protease SAG12-like [*Brassica napus*] / XP_013697317.1 ; senescence-specific cysteine protease SAG12-like precursor [*Brassica napus*] / NP_001302502.1	Senescence-specific cysteine protease SAG12-like [*Brassica napus*] / XP_013697317.1 / 93 %	SAG12-like
